# Mechanisms of Arrhythmogenicity of Hypertrophic Cardiomyopathy-Associated Troponin T (*TNNT2*) Variant I79N

**DOI:** 10.3389/fcell.2021.787581

**Published:** 2021-12-17

**Authors:** Sanam Shafaattalab, Alison Y Li, Marvin G Gunawan, BaRun Kim, Farah Jayousi, Yasaman Maaref, Zhen Song, James N Weiss, R. John Solaro, Zhilin Qu, Glen F Tibbits

**Affiliations:** ^1^ Biomedical Physiology and Kinesiology, Simon Fraser University, Burnaby, BC, Canada; ^2^ Cellular and Regenerative Medicine Centre, BC Children’s Hospital Research Institute, Vancouver, BC, Canada; ^3^ Department of Biochemistry and Molecular Biology, The University of British Columbia, Vancouver, BC, Canada; ^4^ UCLA Cardiac Computation Lab, David Geffen School of Medicine, University of California, Los Angeles, Los Angeles, CA, United States; ^5^ Department of Physiology and Biophysics, University of Illinois at Chicago, Chicago, IL, United States; ^6^ Department of Molecular Biology and Biochemistry, Simon Fraser University, Burnaby, BC, Canada; ^7^ Department of Biomedical Engineering, University of British Columbia, Vancouver, BC, Canada

**Keywords:** human iPSC-derived cardiomyocyte (hiPSC-CM), troponin T, hypertrophic cardiomyopathy, optical mapping of calcium and action potentials, cardiomyocyte calcium

## Abstract

Hypertrophic cardiomyopathy (HCM) is the most common heritable cardiovascular disease and often results in cardiac remodeling and an increased incidence of sudden cardiac arrest (SCA) and death, especially in youth and young adults. Among thousands of different variants found in HCM patients, variants of *TNNT2* (cardiac troponin T—TNNT2) are linked to increased risk of ventricular arrhythmogenesis and sudden death despite causing little to no cardiac hypertrophy. Therefore, studying the effect of *TNNT2* variants on cardiac propensity for arrhythmogenesis can pave the way for characterizing HCM in susceptible patients before sudden cardiac arrest occurs. In this study, a *TNNT2* variant, I79N, was generated in human cardiac recombinant/reconstituted thin filaments (hcRTF) to investigate the effect of the mutation on myofilament Ca^2+^ sensitivity and Ca^2+^ dissociation rate using steady-state and stopped-flow fluorescence techniques. The results revealed that the I79N variant significantly increases myofilament Ca^2+^ sensitivity and decreases the Ca^2+^ off-rate constant (*k*_off_). To investigate further, a heterozygous I79N^+/−^ *TNNT2* variant was introduced into human-induced pluripotent stem cells using CRISPR/Cas9 and subsequently differentiated into ventricular cardiomyocytes (hiPSC-CMs). To study the arrhythmogenic properties, monolayers of I79N^+/−^ hiPSC-CMs were studied in comparison to their isogenic controls. Arrhythmogenesis was investigated by measuring voltage (*V*_m_) and cytosolic Ca^2+^ transients over a range of stimulation frequencies. An increasing stimulation frequency was applied to the cells, from 55 to 75 bpm. The results of this protocol showed that the TnT-I79N cells had reduced intracellular Ca^2+^ transients due to the enhanced cytosolic Ca^2+^ buffering. These changes in Ca^2+^ handling resulted in beat-to-beat instability and triangulation of the cardiac action potential, which are predictors of arrhythmia risk. While wild-type (WT) hiPSC-CMs were accurately entrained to frequencies of at least 150 bpm, the I79N hiPSC-CMs demonstrated clear patterns of alternans for both *V*_m_ and Ca^2+^ transients at frequencies >75 bpm. Lastly, a transcriptomic analysis was conducted on WT vs. I79N^+/−^ *TNNT2* hiPSC-CMs using a custom NanoString codeset. The results showed a significant upregulation of *NPPA* (atrial natriuretic peptide), *NPPB* (brain natriuretic peptide), Notch signaling pathway components, and other extracellular matrix (ECM) remodeling components in I79N^+/−^ vs. the isogenic control. This significant shift demonstrates that this missense in the *TNNT2* transcript likely causes a biophysical trigger, which initiates this significant alteration in the transcriptome. This TnT-I79N hiPSC-CM model not only reproduces key cellular features of HCM-linked mutations but also suggests that this variant causes uncharted pro-arrhythmic changes to the human action potential and gene expression.

## Introduction

Hypertrophic cardiomyopathy (HCM) is a genetic disorder that presents clinically as ventricular and/or septal hypertrophy, myocyte disarray, and increased myocardial fibrosis (Maron et al., 1995; Maron et al., 2006). It is a relatively common (1:500) autosomal dominant disease, making it the most common genetic cardiovascular disease in many countries (Maron et al., 1995). The structural changes that occur in this disorder are often clinically asymptomatic, yet their presence can precipitate sudden cardiac arrest (SCA) and death as the first overt manifestation due to their strong arrhythmogenicity. HCM is the most common cause of the SCA in young people, affecting 1%–2% of children and adolescents and up to 1% of young adults in HCM community cohorts (Elliott et al., 2000; Maron, 2002). Since the seminal finding of a β-myosin heavy chain (*MYH7*) gene mutation in 1995, more than 1,000 different variants have been identified in over 20 HCM candidate genes (Tester and Ackerman, 2009), most of which code for sarcomeric proteins.

Variants found in the cardiac troponin T gene (*TNNT2*) are the third most common cause of HCM after β-myosin heavy chain and myosin binding protein-C (*MYBPC3*) (Messer et al., 2016). The HCM-linked troponin T mutations can induce a clinical HCM phenotype, increase myofilament Ca^2+^ sensitivity, and cause arrhythmogenesis (Knollmann and Potter, 2001). Unlike the general anatomical abnormalities normally found in HCM patients, hearts from patients harboring cardiac troponin T (*TNNT2*) variants typically show significantly less ventricular hypertrophy compared to other HCM-associated variants. This low degree of clinical penetrance of the *TNNT2* variants makes them difficult to detect by echocardiography (Watkins et al., 1995; Knollmann and Potter, 2001; Tadros et al., 2020), yet these patients are at a high risk of SCA. This suggests that the degree of hypertrophy does not necessarily correlate with the risk of SCA and that other mechanisms likely contribute to the devastating outcomes for patients with *TNNT2* variants (Knollmann and Potter, 2001).

The separation of degree of hypertrophy from the risk for SCA was shown clearly in a series of seminal studies done by the Knollmann group in which transgenic mice expressing I79N, F110I, and R278C *TNNT2* variants were studied prior to the development of any overt form of hypertrophy (Knollmann et al., 2003; Baudenbacher et al., 2008; Schober et al., 2012; Eschenhagen et al., 2015). Mice expressing the I79N variant were particularly susceptible to ventricular tachycardia and arrhythmias that was coincident with an increased myofibrillar Ca^2+^ sensitivity, action potential triangulation, and increased dispersion of ventricular conduction velocities at fast heart rates in the absence of structural abnormalities (Baudenbacher et al., 2008; Schober et al., 2012). These results with I79N *TNNT2* in transgenic mice were subsequently corroborated by the same group using human induced pluripotent stem cell-derived cardiomyocytes (hiPSC-CMs) (Wang et al., 2018).

In this study, we investigated the altered Ca^2+^-binding properties and the propensity for arrhythmias induced by a *TNNT2* mutation, I79N, in human cardiac reconstituted thin filament (hcRTF) system and hiPSC-CMs.

Both human recombinant cardiac troponin (cTn) complexes and hcRTF were used to determine the changes in Ca^2+^ sensitivity and dissociation using steady-state and stopped-flow fluorescence techniques, respectively.

In addition, we used hiPSC-CMs, which have been used to model a variety of inherited arrhythmias and cardiomyopathies (Sun et al., 2012; Eschenhagen et al., 2015; Shafaattalab et al., 2019; Paige et al., 2020). These hiPSC-CMs robustly express contractile proteins including troponin I and troponin T (Hwang et al., 2015; Dewar et al., 2017; Shafaattalab et al., 2019). Our hiPSC-CMs express the adult form of cardiac TnT at a higher level than the fetal form and, therefore, are an ideal model to study the effect of variants in the adult form of troponin T. In the present study, we used the CRISPR/Cas9 genome-editing tool to generate heterozygous I79N^+/−^ *TNNT2* hiPSC-CMs.

It is generally accepted that HCM-causing genetic variants increase the Ca^2+^ sensitivity of the contractile element. Among the many different HCM-causing mutations, *TNNT2* variants can be considered as one of the most critical, since they have a low degree of clinical penetrance. Studies in mice suggest that the HCM-associated TnT-I79N variant increases myofilament Ca^2+^ sensitivity and is highly arrhythmogenic, but whether findings from mice translate to human cardiomyocyte electrophysiology was not as well established. This is where the necessity of using human models to further investigate the I79N variant emerges.

In this study, we used the hcRTF system and human-induced pluripotent stem cell-derived cardiomyocytes to investigate the altered Ca^2+^-binding properties and the propensity for arrhythmias induced by a *TNNT2* variant, I79N. Based on the modeling data, the arrhythmogenicity is related, at least in part, to the reduced off-rate constant (*k*_off_) of Ca^2+^ from the myofilaments. Action potential triangulation, which is purported to be arrhythmogenic, was clearly visible at low stimulation frequencies in I79N^+/−^ hiPSC-CMs but was non-visible at higher frequencies, which elicited alternans. In addition, the mRNA expression profile strongly indicates that this *TNNT2* variant induces a major remodeling response even with the cells not being fully mature, a finding which deserves further exploration.

## Methods

### Generation of Human Cardiac Reconstituted Thin Filaments

The adult cTn complexes were generated from recombinant human adult troponin subunits (expressed from *TNNC1*, *TNNI3*, and *TNNT2*). Reconstituted thin filaments were generated by mixing each cTn complex with human alpha-tropomyosin (*TPM1*) as well as native actin extracted from rabbit skeletal muscle, which has a 99.2% sequence identity with human cardiac actin (Dewar et al., 2017). See Supplementary Table S1 for the complete list of the constructs utilized in generating the hcRTF.

### Determination of hcRTF Ca^2+^ Kinetics

All steady-state fluorescence measurements were performed using a Cary Eclipse fluorescence spectrophotometer (Agilent) at 15 ± 0.1°C as described (Dewar et al., 2017). In brief, IAANS fluorescence was excited at 330 nm and monitored at the peak of the emission of 450 nm. Microliter amounts of CaCl_2_ were added to 2 ml of each labeled hcRTF in a titration buffer containing the following (in millimolars): 200 MOPS, 150 KCl, 3 MgCl_2_, and 1 DTT at pH 7.0. At least five to eight datasets were collected for each construct, and the data were fit with a sigmoidal fit (based on the Hill equation) in Origin 8.5 (Microcal Software, Northampton, MA). The Ca^2+^ sensitivity for each hcRTF was represented by the dissociation constant *K*_d_ (±SEM) defined as the Ca^2+^ concentration at the half-maximal fluorescence change. Two means were considered to be significantly different when *p* ≤ 0.05.

Ca^2+^ dissociation rates [*k*_off(Ca_^2+^_)_] from each hcRTF were characterized using a Chirascan stopped-flow apparatus (Applied Photophysics, Surrey, United Kingdom) with a dead time of 1.1 ms at 15.0 ± 0.1°C. IAANS fluorescence was excited at 300 nm and monitored through a 510-nm broad band-pass interference filter (Semrock, Rochester, NY). Each trace was obtained as the solution containing hcRTF saturated with 200 μM CaCl_2_ was rapidly mixed with 10 mM EGTA solution in the stopped-flow buffer containing the following(in millimolars): 10 MOPS, 150 KCl, 3 MgCl_2_, and 1 DTT at pH 7.0. Each *k*_off(Ca_^2+^_)_ value represents an average of at least five traces fit with a single exponential equation and repeated more than 15 times. Two means were considered to be significantly different when *p* ≤ 0.05.

### Human iPSC Maintenance and Cardiomyocyte Differentiation

Human iPSC lines (iPS IMR90-1) were obtained from the Wicell Research Institute (https://www.wicell.org/). Differentiation of hiPSCs to cardiomyocytes (hiPSC-CMs) employed a protocol published previously (Dewar et al., 2017). In brief, hiPSCs were maintained on Corning Matrigel (0.5 mg/six-well plate, dissolved in DMEM/F-12 medium) cultured in mTeSR1 medium (StemCell Technologies, Vancouver, BC). Cells were passaged every 4 days using Versene solution (Thermo Fisher, Waltham, MA) and were then seeded on a six-well Matrigel-coated plate at a density of 100,000 cells cm^−2^ in mTeSR1 medium. The medium was changed daily, and after 3–4 days, when the monolayer of cells reached >90% confluency, the mTeSR1 medium was replaced with Roswell Park Memorial Institute (RPMI) 1640 basal medium (Thermo Fisher, Waltham, MA) plus B27 without insulin supplement (Thermo Fisher) containing 12 µM CHIR99021 (R&D Systems) for 24 h, followed by 5 mM IWP4 (Tocris) for 2 days without medium change. The medium was changed to RPMI 1640 plus B27 complete supplement, and then subsequently, the medium was changed every 2–3 days. Robust spontaneous beating of the monolayer was observed by day 12.

### Preparation and Design of CRISPR-Cas9 and Donor Template

Single-guide RNAs (sgRNA) were designed to target the region close to I79 *TNNT2* using online CRISPR tools including CRISPRko Tool from the Broad Institute (Nivala et al., 2012a), the CRISPR Finder from Wellcome Trust Sanger Institute (Nivala et al., 2015), and Benchling (Mahajan et al., 2008). The sgRNA sequences used in the study are shown in Supplementary Table S2. The sgRNAs were cloned into a pCCC vector, which is based on the pSpCas9(BB)-2A-GFP vector (PX458, Addgene plasmid # 48,138) as detailed previously (Marston and Zamora, 2020; Menon et al., 2008). The pCCC vector contains the complete U6 promoter for enhanced expression in hiPSCs. The 127-bp asymmetric single-stranded donor nucleotides (ssODNs) (Marston and Zamora, 2020; Menon et al., 2008; Pasquale et al., 2012) were designed (Supplementary Table S2) to include the transition (T → A) that is seen in I79N, as well as a silent mutation at the PAM site to prevent the continuous cutting and improve the efficiency of the HDR repair (Pasquale et al., 2012).

### Transfection and Fluorescence-Activated Cell Sorting

Human iPSCs were co-transfected with a PX458 plasmid and ssODN using Lipofectamine 3000 (Thermo Fisher). Briefly, 2 × 10^5^ hiPSCs were transfected with 500 ng sgRNA and 10 pmol of ssODN. Since the vector contained GFP as a reporter, the GFP^+^ hiPSCs were sorted as single cells using fluorescence-activated cell sorting (FACS) 48–72 h post-transfection and were plated on Matrigel-coated plates in mTeSR1 (Dewar et al., 2017). More than 100 single-cell colonies were assayed. To screen for on-target single mutation, genomic DNA was extracted and used for PCR amplification of the CRISPR target sites. These amplicons were sequenced to confirm the single-nucleotide mutation at the I79 *TNNT2* site using the primers described in Supplementary Table S2. Genome-edited hiPSC lines were retested for pluripotency and genome integrity as described.

### Optical Mapping for Action Potential and Calcium Transient Recordings

Prior to imaging, a monolayer of hiPSC-CM was perfused with IMDM supplemented with a physiological solution (final concentrations in millimolars): 140 NaCl, 3.6 KCl, 1.2 CaCl_2_, 1 MgCl_2_, 10 HEPES, and 5.5 D-glucose for optical mapping as described (Varnava et al., 2001; Baudenbacher et al., 2008; Dewar et al., 2017). In brief, the cells were incubated with 15 μM of the potentiometric dye RH-237 (Molecular Probes, Eugene, OR) for 45 min. After incubating the hiPSC-CMs with RH-237, 5 μM of the Ca^2+^-sensitive dye Rhod-2AM (Molecular Probes) and 20 μM blebbistatin [a myosin ATPase inhibitor (Sigma-Aldrich)] were added to the culture dish. Blebbistatin inhibits contraction and reduces motion artifact with no detectable effect on electrophysiological properties (Rust et al., 1999). For imaging, the hiPSC-CMs were excited by 532-nm LEDs. RH-237 and Rhod-2 emissions were monitored using a >710-nm long-pass and 565–600-nm band-pass filters, respectively, at 100 fps at 37°C. The spectral properties allow both dyes to be fluoresced using 532 nm and their respective emissions to be separated by dichroic mirrors. Both signals were captured with a single Hamamatsu ORCA-Flash4 digital CMOS camera by incorporating an optical image splitter. Electrical field stimulation was applied using stainless steel electrodes. The electrodes were ∼1 cm apart, which were placed in the imaging chamber. The hiPSC-CMs harboring the *TNNT2* heterozygous mutation I79N^+/−^ and wild type (WT) were stimulated for 20 s each at four stimulation frequencies (55, 65, 75, and 100 beats/min). Data were analyzed using a custom-built software in IDL (Exelis Visual Information Solutions, McLean, VA) (Varnava et al., 2001; Baudenbacher et al., 2008; Dewar et al., 2017).

### Computer Simulation

Computer simulations were carried out to investigate the mechanisms of alternans promoted by the I79Nvariant. We used a ventricular cell model developed previously (Nivala et al., 2012b), consisting of a three-dimension network of 20,000 (100 × 20 × 10) coupled Ca^2+^ release units (CRUs). The model has been used to investigate spatiotemporal Ca^2+^ cycling and action potential dynamics in ventricular myocytes, including the genesis of Ca^2+^ waves and Ca^2+^ alternans (Nivala et al., 2012a; Nivala et al., 2015). Details of the model have been published previously (Nivala et al., 2012b), and the parameters were the same except for the *K*_D_ of SERCA, which was changed from 1.0 to 0.25 μM. In addition, troponin C buffers were added to the model. The formulation is listed below, which is adopted from our previous model (Mahajan et al., 2008):

$$J_{trpn}=k_{on}\left(B_{t}-{\left[CaT\right]}_{i}\right){\left[Ca^{2+}\right]}_{i}-k_{off}{\left[CaT\right]}_{i}$$

where $k_{on}=0.0327{\left(\mu Mms\right)}^{-1}$ and $k_{off}=0.5ms^{-1}$ . To simulate the effects of the I79N^+/−^ variant on Ca^2+^ alternans, the dissociation rate (*k*_off_) of Ca^2+^ from troponin C was reduced to 0.1 ms^−1^.

### mRNA Expression Profile

Quantitative real-time PCR: Total RNA was extracted from hiPCS-CM using TRIzol followed by RNeasy kit (Qiagen, Mississauga, ON), according to the manufacturer’s instructions. The concentration and purity of the RNA was determined using a NanoDrop ND-1000 spectrophotometer (Thermo Fisher Scientific). The cDNA was synthesized using Qiagen Quantitect Reverse Transcription kit, according to the manufacturer’s instructions. Real-time qRT-PCR analysis was performed on a Bio-Rad CFX96 Touch Real-Time PCR System (Bio-Rad, Mississauga, ON). Primers were designed using Primer3 and OligoAnalyzer 3.1. Analysis was performed according to the ΔΔCt method using the geometric mean of housekeeping genes β-actin and GAPDH to normalize the qRT-PCR data.

Multiplexed mRNA profiling was conducted using a custom codeset containing 251 gene probes synthesized by NanoString Technologies Inc. and the NanoString nCounter® SPRINT Profiler28 (see Supplementary Table S3 for a list of all transcripts included in the codeset). A total of 50 ng of purified RNA per sample was hybridized overnight (16 h) to the custom capture and reporter probes. Hybridized samples were loaded into each channel of the nCounter® SPRINT cartridge. Raw mRNA counts were collected, and the results were normalized to seven housekeeping genes (PIK3CA, ATP5F1, IPO8, PPIA, SPCS1, AKT, and RPS13). Analysis was performed on the nSolver analysis software and the Advanced Analysis module (NanoString Technologies Inc.).

### Statistical Analysis

Data were presented as mean ± SEM unless noted otherwise. Statistical analysis and data visualization of hcRTF Ca^2+^ kinetics, optical mapping, and NanoString data were conducted using R version 3.6.1. Unpaired Student’s *t* tests were conducted to compare two groups (WT vs I79N^+/−^ *TNNT2* hiPSC-CMs) in all analysis except for the differential expression of genes (DEG) analysis of the NanoString data. For DEG, the fold-change of each gene was compared between the WT and I79N^+/−^ *TNNT2* hiPSC-CMs using Welch’s *t*-test and was corrected for multiple comparisons using the Benjamini–Yekutieli method to calculate the false discovery rate (FDR). A *p* value <0.05 was considered statistically significant with the following notation: *p < 0.05, **p < 0.01, and ***p < 0.001.

## Results

### Sequence Alignment of the TNNT2 N-Terminal Protein Among Different Species

Supplementary Figure S1 compares human *TNNT2* from residues 1 to 109, which are homologous sequences from mammals to teleosts. The conservation of the isoleucine at residue 79 represents the fact that is preserved for more than 400 million years of evolution.

### Ca^2+^ Sensitivity and Dissociation Rates of the Human Cardiac Reconstituted Thin Filaments

**FIGURE 1:**
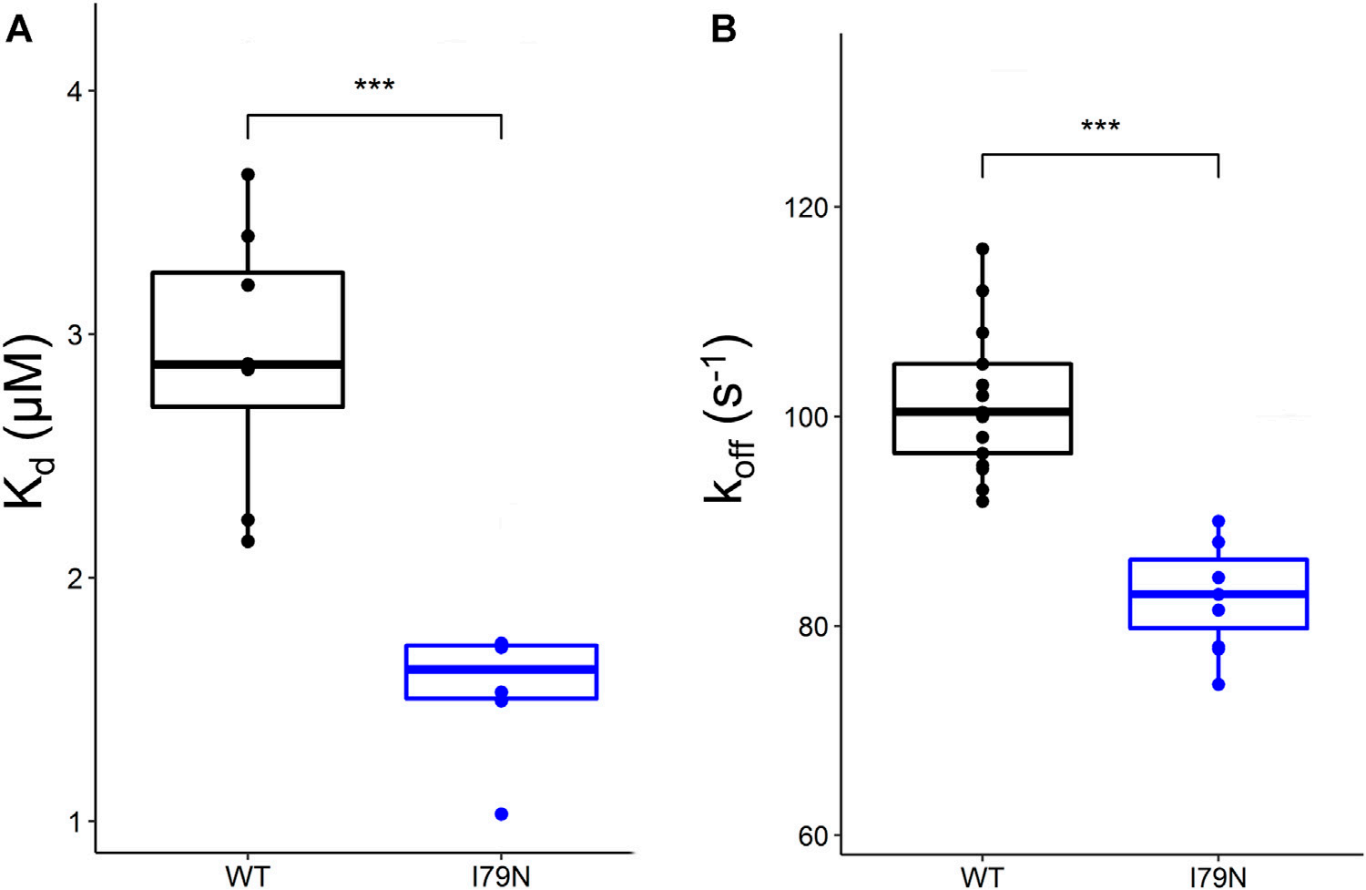
Steady-state and stopped-flow analysis of Ca^2+^ kinetics in human cardiac reconstituted thin filaments (hcRTF). **(A)** The Ca^2+^ sensitivity [expressed in *K*_d_ (μM)] of the wild-type (WT) (*n* = 8 technical replicates) and I79N hcRTF (*n* = 6 technical replicates) was determined by steady-state fluorescence spectrometry. The I79N significantly decreased the *K*_d_ compared to the WT counterpart by ∼1.7-fold, indicating that these variants increase the Ca^2+^ sensitivity significantly in the hcRTF biochemical system. **(B)** A stopped-flow instrument was used to determine the Ca^2+^ dissociation rates (expressed as *k*_off_) from the hcRTF. Upon the addition of EGTA, the Ca^2+^ was rapidly removed from troponin C, inducing a conformational change from cTnC within hcRTF in which the fluorescence change was recorded. The I79N hcRTF (*n* = 11 technical replicates) decreased the *k*_off_ by ∼1.2-fold compared to the WT counterpart (*n* = 9 technical replicates), suggesting that the increase in Ca^2+^ sensitivity observed in the steady state is largely due to a slower Ca^2+^ off-loading rate. Unpaired Student’s *t* tests were conducted to compare WT versus I79N hcRTF, and a *p* value <0.05 was considered statistically significant with the following notation: *p < 0.05, **p < 0.01, and ***p < 0.001.

Steady-state fluorescence measurements were carried out on WT and variant hcRTFs, and Ca^2+^-dependent IAANS fluorescence increase was observed for all hcRTF constructs. The I79N variant shows a statistically significant increase of Ca^2+^ sensitivity by∼2.3-fold (*p* ≤ 0.05) (Figure 1A).

Stopped-flow fluorescence spectroscopy was used to determine the Ca^2+^ dissociation rates from the hcRTF constructs. Upon the addition of EGTA, the Ca^2+^ rapidly dissociated from cTnC, inducing a cTnC conformational change in which the fluorescence change was recorded. As Ca^2+^ was stripped from cTnC by EGTA, a decrease in IAANS fluorescence was observed for all hcRTF constructs. The hcRTF harboring the I79N TnT variant show a significant decrease of *k*_off_ compared to WT, increasing the Ca^2+^ sensitivity by ∼1.4-fold (*p* ≤ 0.05) (Figure 1B). This explains, at least in part, the increase of Ca^2+^ sensitivity conferred by the hcRTF harboring this TNNT2 variant in the steady-state experiments.

### hiPSC-CM Experiments

The NanoString data showed that the expression differences in the *TNNI3*/*TNNI1* ratio were not significantly different between I79N^+/−^ and WT lines (Supplementary Figure S3).

To understand how the I79N^+/−^ *TNNT2* variant impacts cardiomyocyte function, genome-edited I79N^+/−^ *TNNT2* hiPSCs were generated and differentiated to cardiomyocytes. The heterozygous 179N^+/−^ *TNNT2* hiPSC colonies were identified by Sanger sequencing (Supplementary Figure S2). The I79N^+/−^ *TNNT2* hiPSCs did not have any off-target mutations for the 10 most homologous sites predicted by the Broad Institute algorithm (Nivala et al., 2012a).

**FIGURE 2:**
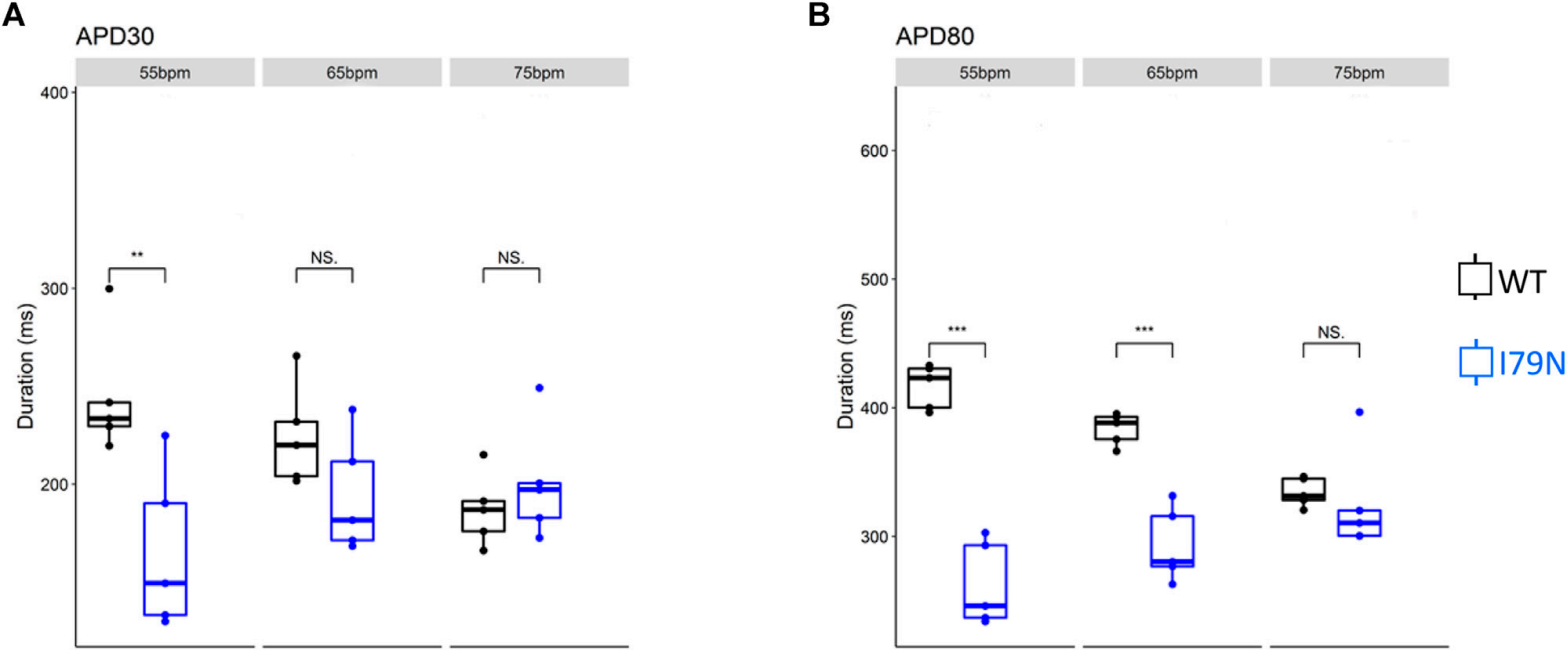
Influence of stimulation frequency on action potential duration in WT (black) and I79N^+/−^ (blue) *TNNT2* hiPSC-CMs. **(A, B)** The data at APD_30_ and APD_80_, respectively. The box plots exhibit the impact of increasing the stimulation frequency from 55 to 75 bpm on these parameters. The two groups, WT vs. I79N^+/−^, were compared using unpaired Student’s *t* tests with *n* = 5 biological and *n* = 2 technical replicates per condition. A *p* value <0.05 was considered statistically significant with the following notation: *p < 0.05, **p < 0.01, and ***p < 0.001.

**FIGURE 3:**
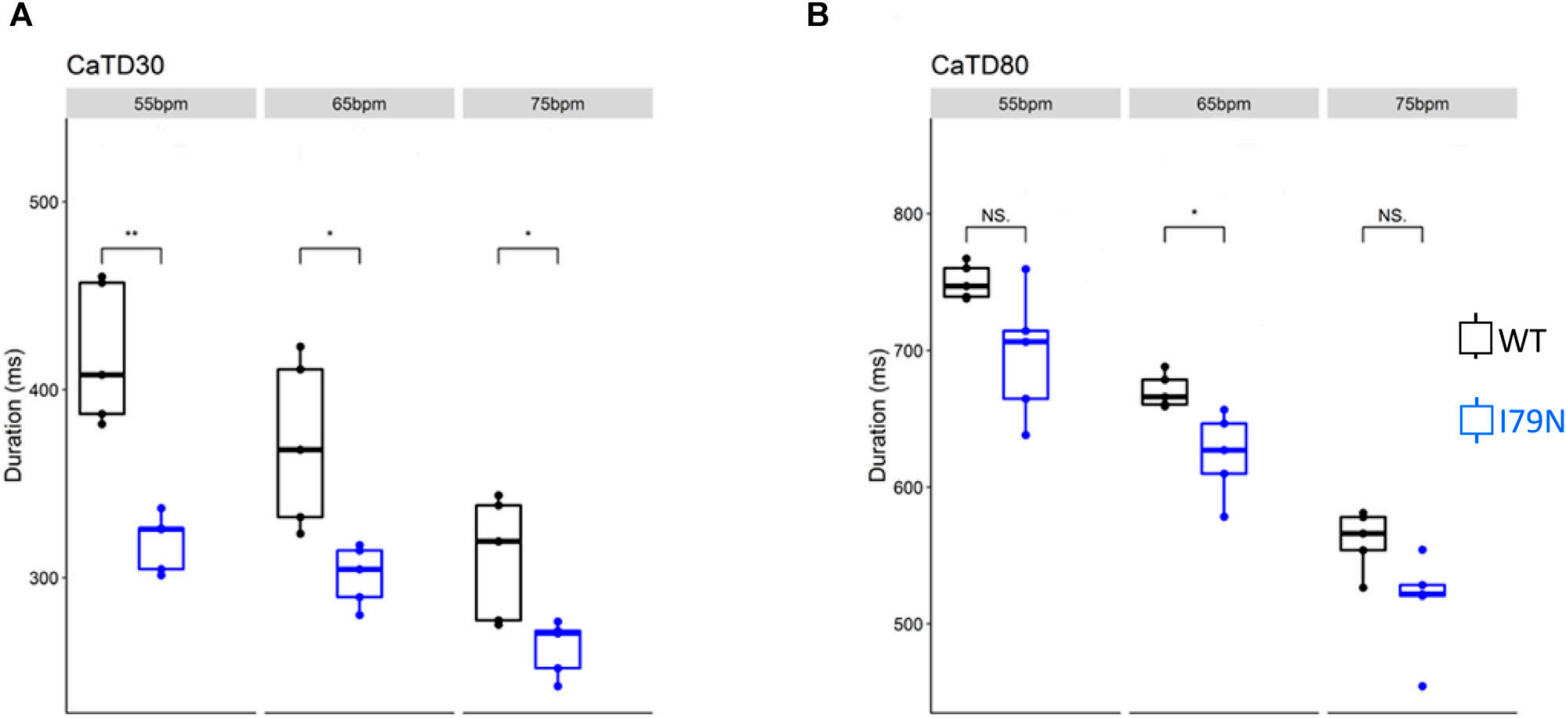
The influence of stimulation frequency on Ca^2+^ transient duration in WT (black) and I79N^+/−^ (blue) *TNNT2* hiPSC-CMs. **(A, B)** The data at Ca^2+^ transient durations of CaTD_30_ and CaTD_80_, respectively. The box plots exhibit the impact of increasing the stimulation frequency from 55 to 75 bpm on these parameters. I79N^+/−^ hiPSC-CMs exhibited a significantly shorter CaTD_30_ than WT under all conditions and significantly shorter CaTD_80_ at 65 bpm. The two groups, WT vs. I79N^+/−^, were compared statistically using unpaired Student’s *t* tests with *n* = 5 biological and *n* = 2 technical replicates per condition. A *p* value <0.05 was considered statistically significant with the following notation: *p < 0.05, **p < 0.01, and ***p < 0.001.

We next investigated the effect of various stimulation frequencies in WT and I79N^+/−^ hiPSC-CMs. Membrane voltage (*V*_m_) and cytosolic Ca^2+^ transients were determined from the fluorescence emitted by RH-237 and Rhod-2, respectively. Figure 2 shows the duration of the *V*_m_ transients from hiPSC-CM monolayers stimulated at 55, 65, and 75 bpm. Figure 2A shows the action potential duration at 30% repolarization (APD_30_), and Figure 2B shows the APD_80_ as a function of stimulation frequency. Figure 3A shows the Ca^2+^ transient duration at 30% recovery (CaTD_30_), and Figure 3B depicts the CaTD_80_ as a function of frequency. The *V*_m_ and Ca^2+^ transients from both WT and I79N^+/−^ hiPSC-CMs entrained normally to 55, 65, and 75 bpm stimulation frequencies. These frequencies were chosen because 55 bpm was higher than the intrinsic beating rate of all hiPSC-CMs and 75 bpm was below frequencies that induced any observable form of arrhythmia. The action potential morphology showed significant triangulation [(APD_80_ − APD_30_) / APD_80_] in I79N^+/−^ compared to WT hiPSC-CMs at 55 but not at 65 and 75 bpm (data not shown).

**FIGURE 4:**
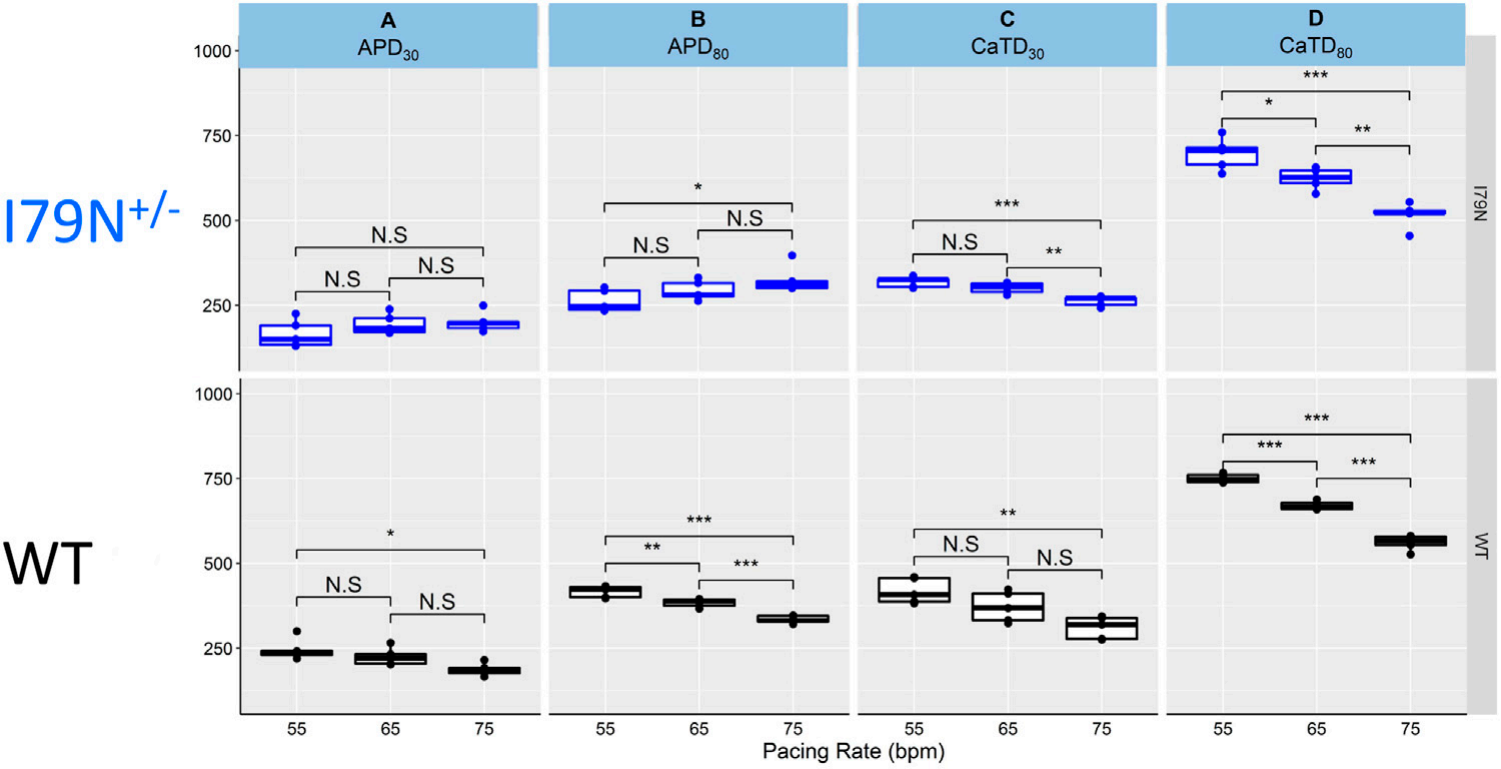
The influence of stimulation frequency on action potential duration (APD) and Ca^2+^ transient duration in WT (black) and I79N^+/−^ (blue) *TNNT2* hiPSC-CMs. **(A, B)** The analysis of these data at APD_30_ and APD_80_, respectively. The box plots exhibit the impact of increasing the stimulation frequency from 55 to 75 bpm on the APD_30_ and APD_80_, respectively. The box extends from the first to the third quartile; the line within the box is the median, and the downward and upward whiskers are the minimum and maximum data points, respectively. **(C, D)** The analysis of these data at CaTD_30_ and CaTD_80_, respectively. The box plots exhibit the impact of increasing the stimulation frequency from 55 to 75 bpm on the CaTD_30_ and CaTD_80_. The box extends from the first to the third quartile; the line within the box is the median, and the downward and upward whiskers are the minimum and maximum data points, respectively. The data from the two groups, WT vs. I79N^+/−^, were compared statistically using one-way ANOVA with Tukey test for multiple comparison correction with *n* = 5 biological and *n* = 2 technical replicates. Annotations are N.S for not significant, *p < 0.05, **p < 0.01, and ***p < 0.001.

Figure 4 shows the same data as in Figures 2 and 3 but allows for a direct comparison of each parameter across the spectrum of frequencies. While WT hiPSC-CMs show a significant reduction of APD_30_ and APD_80_ as the frequency is increased from 55 to 75 bpm, the same does not hold true for I79N^+/−^ hiPSC-CMs. In contrast, the Ca^2+^ transient duration decreases significantly in both groups with increased stimulation frequency.

**FIGURE 5:**
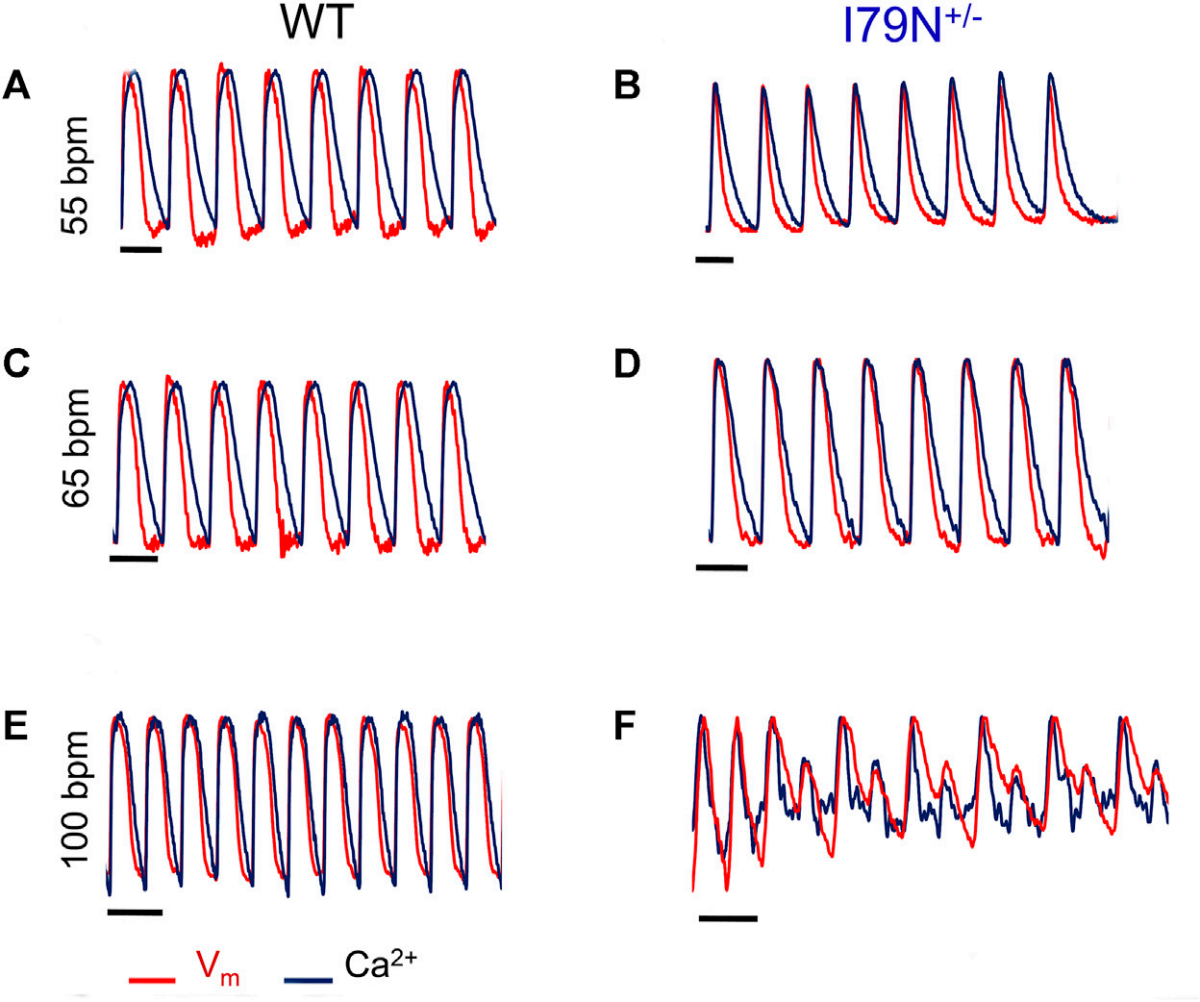
Representative optical mapping traces of voltage (*V*_m—_red) and Ca2+ transients (blue) in hiPSC-CMs stimulated at different frequencies. Monolayers of hiPSC-CMs WT and I79N^+/−^ *TNNT2* were loaded with voltage- (RH-237) and Ca^2+^-dependent (Rhod-2AM) dyes and optically mapped while being field stimulated at 55 bpm **(A, B)**, 65 bpm **(C, D)**, and 100 bpm **(E, F)** for 20 s each. The *TNNT2* WT hiPSC-CMs exhibited clear and significant shortening in the duration of both the action potential and Ca^2+^ transients as a function of stimulation frequency [**(A)** vs. **(E)]** while I79N^+/−^ hiPSC-CMs did not. Furthermore, as the stimulation frequencies reached 100 bpm, the hiPSC-CMs harboring the I79N^+/−^ variant clearly displayed irregular voltage and Ca^2+^ transients **(F)**, which were never observed in WT hiPSC-CMs. Moreover, eight irregular voltage and Ca^2+^ transients were recorded over the 20-s interval (*n* = 5 biological and *n* = 2 technical replicates for each group).

Representative optical mapping traces of *V*_m_ (red) and Ca^2+^ transients (black) at stimulation frequencies of 55, 65, and 100 bpm are shown in Figure 5. Both WT and I79N^+/−^ hiPSC-CMs entrained well to frequencies up to 75 bpm. However, at stimulation frequencies greater than 75 bpm, the arrhythmogenicity of the I79N^+/−^ lines is clearly demonstrated with an alternans pattern seen for both voltage and Ca^2+^ transients. In stark contrast, WT hiPSC-CMs could be entrained normally to stimulation frequencies >150 bpm (data not shown).

**FIGURE 6:**
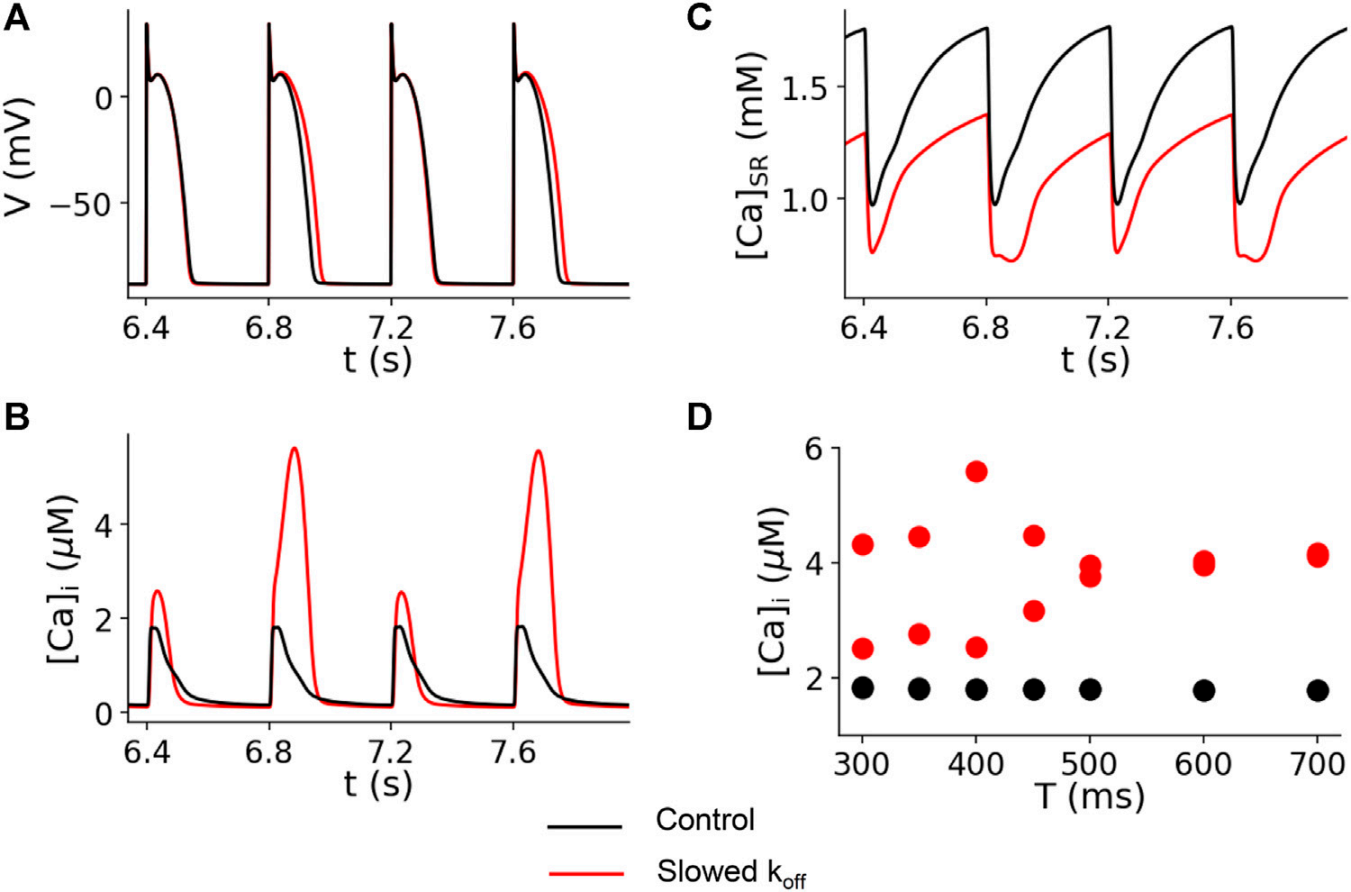
Computer simulation showing that reducing the *k*_off(Ca_^2+^_)_ promotes Ca^2+^ alternans. Time traces of membrane voltage **(A)**, cytosolic free Ca^2+^ **(B)**, and SR luminal free Ca^2+^ **(C)** with both the control (*k*_off_ = 0.5 ms^−1^, black) and a slowed (*k*_off_ = 0.5 ms^−1^, red) Ca^2+^-troponin C unbinding rate. PCL = 400 ms. **(D)** Bifurcation diagrams for both the control (black dots) and a slowed (red dots) unbinding rate, plotting the peak cytosolic Ca^2+^ concentrations of the last two beats against PCL. For the control case, there is only one Ca^2+^ peak value for each PCL for the two recorded beats, indicating the absence of alternans. For the slowed binding rate case, when the PCL is shorter than 500 ms, there are two Ca^2+^ peak values for each PCL for the two recorded beats, indicating the presence of alternans.

The computer simulation results for the control (*k*_off_ = 0.5 ms^−1^) and slowed (*k*_off_ = 0.1 ms^−1^) Ca^2+^ off rate constants are shown in Figure 6. This figure shows the simulated traces for voltage (Figure 6A), cytosolic Ca^2+^ concentration (Figure 6B), and sarcoplasmic reticulum (SR) Ca^2+^ concentration (Figure 6C) for paced cycle length (PCL) = 400 ms. The modeling shows no alternans for the control dissociation rate (black traces), but alternans occurred for the reduced off rate (red traces). In Figure 6D, we plot the peak Ca^2+^ concentration versus PCL. For the control experimental conditions (*k*_off_ = 0.5 ms^−1^), no alternans was present for PCLs from 300 to 700 ms (black dots in Figure 6D) or 86–200 bpm. However, for the reduced *k*_off(Ca_^2+^_)_, alternans occurred when the PCL was shorter than 500 ms (>120 bpm) (red dots in Figure 6D).

To characterize further the effects of the I79N^+/−^ *TNNT2* variant, we profiled the gene expression of the variant in comparison to WT using a custom NanoString codeset. Out of the 251 genes in the codeset (Supplementary Table S3), 236 genes were detected above the threshold as determined by the digital counts of the negative controls of the nCounter assay. The unsupervised hierarchical clustering analysis displayed the unbiased similarity profile between the hiPSC-CM samples, namely, the clear segregation between I79N^+/−^ *TNNT2* and WT *TNNT2* hiPSC-CMs (Supplementary Figure S4A, B). The heatmap showed clear patterns of dysregulation in a series of genes between I79N^+/−^ and WT samples. This difference in profile was further reflected in the unsupervised principal component analysis (PCA) showing a strong clustering to *TNNT2* genotype. In PCA, the hiPSC-CM samples were clustered according to the *TNNT2* genotype with samples possessing the I79N^+/−^ or WT *TNNT2* being strongly associated to samples of the same *TNNT2* genotype. The principal component 1 (PC1) and PC2 accounted for 73% and 16% of the total variance, respectively. The high variance observed in PC1 demonstrated that the *TNNT2* mutation variability (i.e., I79N^+/−^ and WT) is the largest driver of the difference at the gene expression level.

**FIGURE 7:**
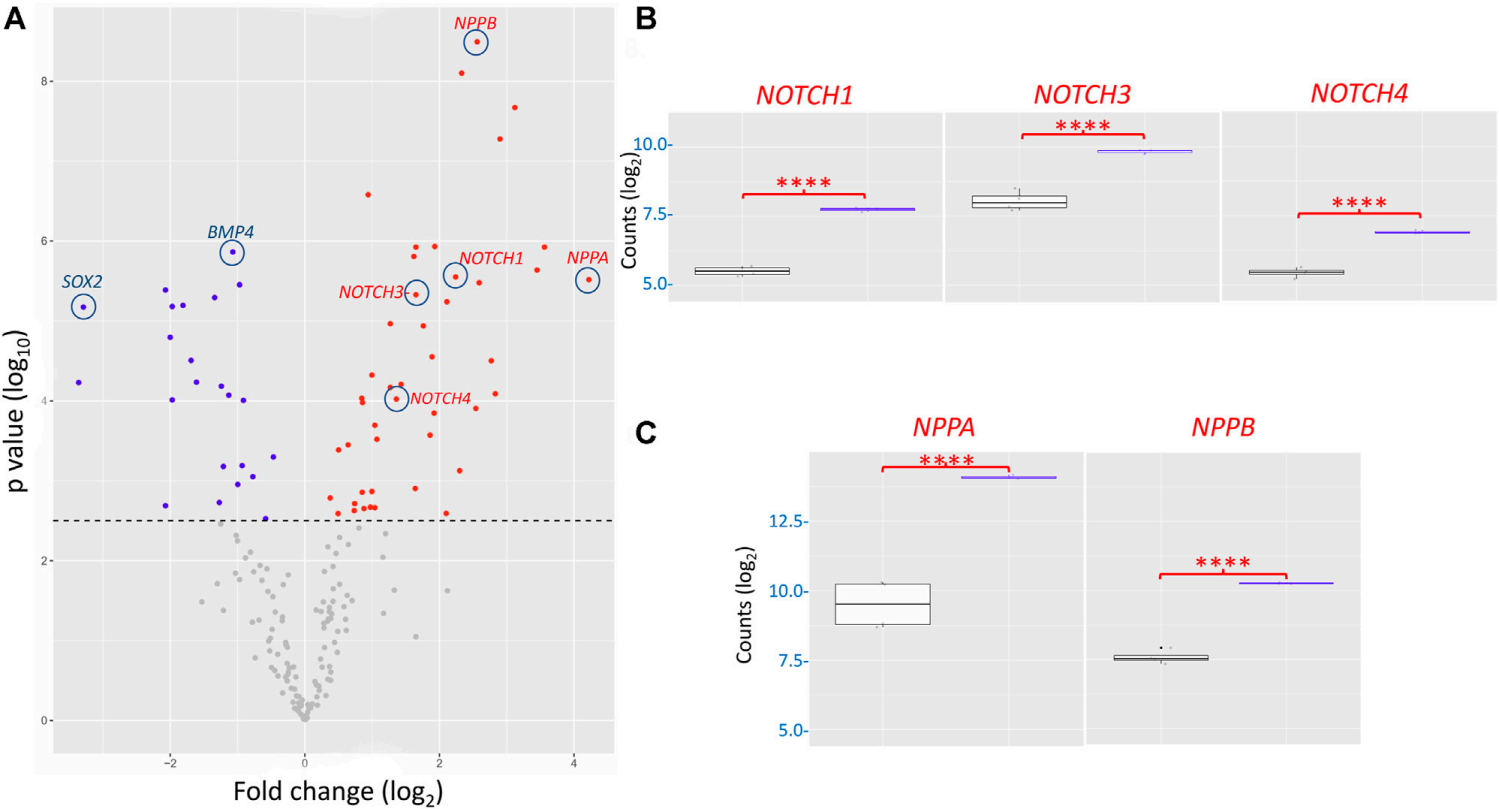
Transcriptomic analysis of the impact of the *TNNT2* I79N^+/−^ variant vs WT using a custom Nanostring codeset of 251 transcripts. **(A)** Volcano plot of I79N vs WT *TNNT2* transcriptomic analysis. From 68 differentially expressed genes (DEG) (adjusted *p* < 0.05), 23 were downregulated (blue) and 45 were upregulated (red). **(B)** The significantly upregulated NOTCH signalling pathway transcripts are shown individually shown in box plots. **(C)** The significantly upregulated cardiac hypertrophy markers ANP (NPPA) and BNP (NPPB) are shown in box plots. For each gene count, the data were normalized against the housekeeping genes, and are presented in log2 scale. The fold-change of each gene was compared between the WT (*n* = 4) vs. I79N^+/−^ (*n* = 3) *TNNT2* hiPSC-CMs using Welch’s *t*-test and was corrected for multiple comparisons using the Benjamini–Yekutieli method to calculate the false discovery rate (FDR). ****p < 0.001.

DEG analysis, illustrated *via* the volcano plot, revealed 68 genes differentially expressed (adjusted *p*-value ˂0.05 with no fold change cut-offs) between the I79N^+/−^ variant and WT hiPSC-CMs (Figure 7A). In the volcano plot, data are presented as the change in the magnitude of gene expression in I79N vs WT, which is shown on the abscissa and probability of the differences being significantly shown on the ordinate. We observed 45 significantly upregulated transcripts and 23 downregulated transcripts in I79N^+/−^, which were respectively shown in red and blue data points of Figure 7A. Highlighted in the volcano plot among the transcripts significantly upregulated in I79N^+/−^ vs. WT are *NPPA* (18.6-fold), which encodes for atrial natriuretic peptide, and *NPPB* (5.9-fold), which encodes for brain natriuretic peptide. In addition, Notch signaling components (*NOTCH1*, *3*, and *4*; *JAG1*; and *HEY1*) were significantly (*p* < 0.001) upregulated in I79N^+/−^ compared to WT. Transcripts encoding for the components of collagen (*COL1A1*, *COL3A1*, and *COL9A2*) were also dramatically upregulated in I79N^+/−^ hiPSC-CMs. Significantly downregulated transcripts included *SOX2* and cardiac developmental marker *BMP4*. The ratio *of TNNI3/TNNI1*, which is used as an index of hiPSC-CM maturation, was 6.1 and 5.6 in I79N^+/−^ and WT, respectively, and was not significantly different between the groups.

We conducted a gene pathway analysis using the Enrichr tool (Chen et al., 2013; Kuleshov et al., 2016) by inputting the list of DEGs between the I79N^+/−^ and WT samples. The top five functional pathways that were found to be significantly dysregulated (adjusted *p*-value ˂0.05) across six gene set libraries are highlighted in Supplementary Figure S5A, B. The most affected pathways in I79N^+/−^ include Notch signaling, both canonical and non-canonical, which includes the maturation precursor as well as the Notch receptor-ligand signaling (Supplementary Figure S5A). The pathways involved in NFAT signaling in cardiac hypertrophy, calcium signaling, heart development, cardiac progenitor differentiation, and cardiac conduction were found to be dysregulated by the mutation as well (Supplementary Figure S5B). Furthermore, the top five enriched Gene Ontology (GO) terms across six GO libraries revealed several mutation-related Notch diseases known to cause congenital heart defects along with atrial fibrillation (AF) and cardiac conduction system to be most significantly (adjusted *p*-value <0.05) associated to 45 upregulated DEGs in I79N^+/−^ (Supplementary Figure S5C). Whereas, 23 downregulated transcripts in I79N^+/−^ were significantly (adjusted *p*-value <0.05) enriched in terms associated with the regulation of cardiac muscle cell membrane repolarization (Supplementary Figure S5D).

## Discussion

Despite over 20 years of studies conducted on HCM-causing *TNNT2* variants, there is an unmet need to understand the connection between biophysical triggers and disease progression. In this study, we focused on the missense TNNT2 mutation I79N^+/−^, which has been associated with a high rate of sudden cardiac death. hcRTF and hiPSC-CMs were used to assess the effects of the I79N variant on Ca^2+^ dissociation kinetics, regulation of excitation/relaxation (voltage and Ca^2+^ transients), and the changes in transcriptome. Our results showed that I79N caused Ca^2+^ mishandling patterns that are consistent with proarrhythmic signals and dysregulated transcriptomic signaling pathways, which further elucidated the cellular mechanisms behind complex cardiomyopathy phenotypes of I79N that lead to sudden cardiac death.

Due to the highly flexible nature of *TNNT2*, most regions remain unsolved except for regions including residues 199–272 that is seen in the high-resolution crystal structure (PDB: 4Y99) (Marston and Zamora, 2020). However, 90% of the cardiomyopathy-causing *TNNT2* variants occur in the unresolved regions, including the I79N *TNNT2* variant. Therefore, it was not possible to carry out computer simulations (e.g., molecular dynamics) for I79N due to the lack of the structural information in this region of the molecule.

Patients harboring the I79N^+/−^ *TNNT2* variant are associated with a high rate of sudden cardiac death at a young age (Thierfelder et al., 1994; Watkins et al., 1995; Menon et al., 2008; Pasquale et al., 2012). Studies on transgenic mice demonstrated that I79N significantly increases the myofilament Ca^2+^ sensitivity, resulting in an increase in susceptibility to ventricular arrhythmia (Baudenbacher et al., 2008), an increase in end-diastolic Ca^2+^ concentration at fast pacing rates, and enhanced SR Ca^2+^ content and release (Schober et al., 2012). These studies were conducted at an age when the mice showed no overt signs of hypertrophy. This group has suggested that due to the fact that the I79N variant increased cytosolic Ca^2+^ buffering, Ca^2+^ may stay bound even at the end of diastole. Our stopped-flow kinetic data on the I79N hcRTF directly supports their speculation, in which the Ca^2+^ dissociation rate was found to be significantly prolonged in the presence of the mutation. When the heart slows, the excess Ca^2+^ bound to the myofilaments during the preceding beats may be re-sequestered back into the SR. This increases the Ca^2+^ release from SR for the next beat, increasing the likelihood of Ca^2+^ being removed from cardiomyocytes by the sodium–calcium exchanger (NCX), potentially resulting in triggered activity or delayed after depolarization. In addition, a heterozygous I79N variant was found in a family with nine affected members and caused different phenotype characteristics of RCM, HCM, DCM, and mixed cardiomyopathy within the same family (Menon et al., 2008). This suggests that this *TNNT2* variant can result in a complex cardiomyopathy with a great diversity of morphological, functional, and clinical features that are potentially caused by different cellular mechanisms arising from other *TNNT2* variants.

### Studies Using Human-Induced Pluripotent Stem Cell-Derived Cardiomyocytes

*TNNT2* variants that cause HCM have been studied in transgenic mice, which have different electrophysiological properties compared to humans (Knollmann et al., 2003). To study *TNNT2* variants in a human model, we used genome editing (CRISPR/Cas9) to generate heterozygous I79N^+/−^ *TNNT2* hiPSCs. This technique enabled us to compare I79N^+/−^ *TNNT2* hiPSCs with its isogenic control hiPSCs instead of comparing with control hiPSCs that have different genetic and epigenetic backgrounds. It has been shown that I79N-related HCM cases are characterized by mild hypertrophy (Watkins et al., 1995). The I79N^+/−^ *TNNT2* hiPSC-CMs expressed some of the hypertrophic transcription factors (Supplementary Figure S6), which is consistent with clinical findings in patients with troponin T mutations (Varnava et al., 2001). We also examined the effect of I79N^+/−^ *TNNT2* variants on voltage and Ca^2+^ transients at different stimulation frequencies (Figures 2 and 3). We found that I79N^+/−^ *TNNT2* hiPSC-CMs exhibited altered Ca^2+^ handling (Figure 2) and shortened early repolarization (Figure 4) and resulted in AP triangulation (Figure 5). In our study, AP triangulation refers to the repolarization time from APD_30_ relative to APD_80_, which is shortened in the *TNNT2* I79N^+/−^ variant (Figures 2 and 3) but only at the lowest stimulation frequencies. *TNNT2* I79N-induced AP triangulation was also observed in a transgenic mouse model by the Knollmann group (Baudenbacher et al., 2008). Here, we demonstrate that this HCM-associated TNNT2 variant is pro-arrhythmic by altering the action potential and Ca^2+^ transient decay rate in human iPSC-CMs, which are consistent with the clinical features of this variant. Although the mechanism of arrhythmogenesis is not clear, alterations in Ca^2+^-handling properties are likely involved.

The expression of mutant TnT protein in myofilaments results in structural and functional changes in cardiac cells (Rust et al., 1999). Unlike the transgenic mice model, myofibril disarray was observed in the I79N^+/−^ *TNNT2* hiPSC-CM model, which is important for understanding the underlying mechanism of TnT-linked HCM (Wang et al., 2018). The rapid heart rate in HCM patients carrying *TNNT2* mutations can be arrhythmogenic. In a study on HCM patients who were recipients of implantable cardiac defibrillators (ICDs), the rapid heart rate resulted in atrial fibrillation or sinus tachycardia in over 90% of the arrhythmic episodes (Cha et al., 2007). In another study in children with HCM who were treated to reduce their heart rates, sinus tachycardia was observed in 90% of ventricular fibrillation episodes (Pablo Kaski et al., 2007). The results of these studies are consistent with the high incidence of ventricular tachycardia and sudden cardiac death possibly linked to high Ca^2+^ sensitivity due to *TNNT2* variants (Watkins et al., 1995; Knollmann and Potter, 2001; Van Driest et al., 2003). Our data from I79N^+/−^ *TNNT2* hiPSC-CMs demonstrated that fast stimulating rates can be arrhythmogenic, compared to WT *TNNT2* hiPSC-CM (Figure 3).

Previous studies found that *TNNT2*-linked HCM variants increased Ca^2+^ sensitivity in the cardiac skinned fibers and slowed the Ca^2+^ dissociation rate [*k*_off (Ca_^2+^_)_] from troponin C during diastole (Baudenbacher et al., 2008; Schober et al., 2012; Gomes et al., 2002; Miller et al., 2001) in the murine heart. However, the total number of Ca^2+^-binding sites did not change in *TNNT2* variants. Therefore, increased Ca^2+^ sensitivity due to *TNNT2* variants increases cytosolic Ca^2+^ buffering because the troponin complex accounts for a substantial portion of cytosolic Ca^2+^ buffering (Miller et al., 2001; Bers, 2000). As expected, increased Ca^2+^ buffering and sensitivity caused a reduction in Ca^2+^ transient peak in I79N^+/−^ *TNNT2* hiPSC-CMs (Wang et al., 2018) at physiological heart rates but, surprisingly, did not change the Ca^2+^ transient amplitude in the transgenic mouse model (Schober et al., 2012). During diastole, increased Ca^2+^ sensitivity results in prolongation of Ca^2+^ transient and slower Ca^2+^ decay rate. Unlike transgenic mice expressing I79N *TNNT2*, but consistent with a previous human model (Wang et al., 2018), we also did not observe prolonged Ca^2+^ transients in I79N^+/−^ *TNNT2* hiPSC-CM (Figure 5). However, our data demonstrated a slower Ca^2+^ decay rate in I79N^+/−^ *TNNT2* hiPSC-CMs compared to WT *TNNT2* hiPSC-CMs (Knollmann et al., 2003) (Figure 2).

It has been reported that the Ca^2+^ sensitization of myofilaments causes action potential remodeling and triangulation, which, in turn, result in induction of arrhythmia in I79N transgenic mice (Knollmann et al., 2003). Previous studies on transgenic mice indicate that the increased Ca^2+^ sensitivity due to I79N variant reduces (Ca^2+^)_free_ during systole and leads to a shorter plateau phase in APD. Moreover, the downregulation of the inward rectifier current (*I*_k1_) in I79N transgenic mice contributes to the shortening of the APD (Knollmann et al., 2003). Our findings also demonstrated this fact in I79N^+/−^ *TNNT2* hiPSC-CM. However, one cannot measure the *I*_k1_ current in an optical mapping assay (Figure 5). One possible explanation for AP triangulation due to increased Ca^2+^ sensitivity is NCX activity (Pott et al., 2007; Bögeholz et al., 2015; Wang et al., 2018). The primary activity of the NCX is during the plateau phase of AP by exchanging the Na^+^ ions with one Ca^2+^ ion, which produces an inward current during phases 2 and 3 of AP. Since the (Ca^2+^)_free_ during systole was reduced due to increased Ca^2+^ sensitivity, the activity of the NCX decreased, resulting in a smaller NCX current and a shorter plateau phase (Pott et al., 2007; Bögeholz et al., 2015). The role of NCX in modulating the AP plateau phase due to Ca^2+^-handling alteration is observed in mouse, rat, and human cardiomyocytes (Knollmann et al., 2003; duBell et al., 1991). However, it is important to note that the NCX activity is different among various species, and APD is less dependent on NCX in higher mammals, which limit the application of this potential mechanism (duBell et al., 1991; London, 2001). The AP remodeling and triangulation in I79N^+/−^ *TNNT2* hiPSC-CMs are considered arrhythmogenic (Wang et al., 2018) and can cause re-entrant ventricular tachycardia in mouse and rabbit models (Boersma et al., 2002). However, in this study, while AP triangulation occurred in AP hiPSC-CMs at the lowest stimulation frequency (55 bpm), it was not apparent at higher frequencies. The arrhythmias, however, were only detectable at the higher stimulation frequencies (>75 bpm), indicating that AP triangulation was not, *per se*, the cause of the arrhythmia. The combination of decreased *I*_k1_^12^, slower Ca^2+^ decay rate, and increased cytosolic (Ca^2+^) during fast pacing may lead to arrhythmogenic events.

In previous studies (Nishikimi et al., 2006; Rovetti et al., 2010), we developed a theory for the mechanism of cytosolic Ca^2+^ alternans. It was called it the “3R” theory since Ca^2+^ alternans is promoted by the interactions of random firing of the Ca^2+^ sparks, recruitment of neighboring Ca^2+^ CRUs for firing (spark-induced sparks), and refractoriness of the CRUs. The recruitment is determined by the coupling strength between CRUs, and Ca^2+^ buffering is one of the parameters affecting recruitment (Nivala et al., 2012a). In this mechanism, Ca^2+^ alternans is promoted by enhancement of the recruitment (Rovetti et al., 2010). This theory can provide a plausible mechanism for alternans promoted by the I79N variant observed in the experiments. Since the slowing of the dissociation of Ca^2+^ from the myofilament by the mutants increases the Ca^2+^–troponin C complex, this reduces Ca^2+^ and troponin C binding. In other words, slowing the dissociation rate of Ca^2+^ from the myofilaments effectively reduces the Ca^2+^ buffering effect and thus enhances the recruitment. The enhancement of recruitment then promotes Ca alternans based on the “3R” theory. Note that in the computer simulations shown in Figure 6, reducing the *k*_off(Ca_^2+^_)_ increases the cytosolic Ca^2+^ transient but reduces the SR Ca^2+^ load, indicating that slowing the Ca^2+^ dissociation rate caused more CRUs to fire due to the enhanced recruitment.

We assessed the impact of the I79N^+/−^ *TNNT2* variant on the transcriptome profile demonstrating increased signaling of a hypertrophic state, profibrotic extracellular matrix (ECM) gene network, and the dysregulation of cardiac developmental pathways. The significant consequence of the I79N^+/−^ variant on the transcriptome profile requires further investigation. Every precaution was taken to ensure, to the best of our ability, that the WT and I79N^+/−^ *TNNT2* variant hiPSC-CMs were as similar as possible except for the point mutation. Both lines were exposed to the CRISPR/Cas9 genome-editing protocol with the I79N^+/−^ line undergoing a double-strand break and then homology-directed repair while the WT remain unchanged. The 10 most likely off-target mutation sites were all sequenced to determine that this was not a contributing factor. Both lines were then subjected to identical differentiation protocols in parallel, and RNA was isolated at the same time. Thus, the most likely explanations for the striking observation are the functional consequences of the variant of biophysical aspects of the contractile process and the alterations in hiPSC-CM Ca^2+^ handling due to the decrease in the Ca^2+^ off-rate constant from the myofibrils.

The significant increase in the expression of the transcripts for atrial natriuretic peptide (ANP) and brain natriuretic peptide (BNP) is indicative of a remodeling taking place that likely reflects a hypertrophic response (Nishikimi et al., 2006). Mechanical stress is an important trigger for ANP and BNP production and release from cardiomyocytes (Nishikimi et al., 2006). In a potentially hypertrophic state induced by HCM-associated variants such as I79N, there is a reappearance of ventricular ANP expression in the developing cardiomyocytes, which is recognized as a marker for the induction of the embryonic gene program seen mainly in ventricular hypertrophy (Razeghi et al., 2001; Gardner, 2003). This shift causes a massive fold increase in ANP and BNP mRNA quantities as seen in the I79N cells and is likely reflecting early indicators of a hypertrophic process (Kerkelä et al., 2015).

Critical components of the Notch signaling pathway (*Notch1*, *Notch3*, *Notch4*, *Jag1*, and *Hey1*) were all significantly upregulated in I79N^+/−^ hiPSC-CMs. The Notch signaling pathway plays an important role during development and maintaining homeostasis by regulating cellular apoptosis, proliferation, and differentiation as well as playing an important role in intercellular communication (Zhou and Liu, 2014). Several studies have shown that Notch signaling is involved in counteracting fibrotic remodeling (D'Amato et al., 2016; MacGrogan et al., 2018; Nistri et al., 2017). Upregulation of the Notch 1, 3, and 4 was also observed in hiPSC-CMs cultured on a stiff substrate, which recapitulates a fibrotic condition as shown by Heras-Bautista et al. (2019). Again, the Notch signaling transcriptomic changes likely reflect a change in the biophysical properties of the hiPSC-CMs harboring the I79N^+/−^ *TNNT2* variant. This striking upregulation in Notch-related transcripts is particularly significant because of the novelty and likely significance of the finding. For example, Notch signaling pathways have been shown to change in response to ischemia and myocardial infarction (Nistri et al., 2017). A major component of HCM progression involves altered microcirculation and the microenvironment of the cardiomyocytes, which could be causal for the Notch transcriptional upregulation (MacGrogan et al., 2018). A study by Øie et al. (2010) investigated the alterations in Notch signaling in rats with HF after induction of myocardial infarction and in humans with heart failure. The isolated cardiomyocytes from both rats and humans showed a significant increase in the relative expression levels of Notch receptors in failing vs. non-failing myocardium (Øie et al., 2010). Another major study by Qiao et al. (2017) investigating sick sinus syndrome showed that the persistent activation of Notch alters gene expression of related ion channels and cellular electrophysiology. This shift predisposes the cardiomyocytes to an arrhythmogenic substrate (Qiao et al., 2017). This could be one possible link between Notch signaling and the arrhythmic phenotype seen in HCM patients with the I79Nvariant. This means that there is a future opportunity for treatment by modulating the Notch pathway to ameliorate the HCM phenotype.

The modification of transcripts associated with collagen was also striking and indicative of a remodeling process. Collagen is the main structural protein of the cardiac ECM, which consists of approximately 85% type I collagen, which is responsible for building thick fibers and is important for strength, and 11% type III collagen, which assembles as thin fibers and plays an important role in ECM elasticity. The ECM also includes collagen types IV, V, and VI; fibronectin; laminin; elastin and fibrillin; proteoglycans; and glycoproteins (Exposito et al., 2010). Collagen type I consists of two chains of collagen type I alpha 1 (*COL1A1*) (which was significantly upregulated in I79N^+/−^ hiPSC-CMs) and one chain of collagen type I alpha 2 (*COL1A2*). The *COL3A1* gene, which encodes for the collagen alpha-1 (III) chain, known as the alpha 1 chain of type III collagen, and was increased significantly in this study. Since the ECM network transmits mechanical signals to cardiomyocytes, disruption or excess production and accumulation of the ECM proteins observed in cardiac fibrosis can significantly affect contraction and relaxation as well as the architecture and function of the heart (Ho et al., 2010). Although the functional consequences of these transcriptomic changes are not fully understood, clearly, the HCM-associated *TNNT2* variant I79N^+/−^ induces a broad spectrum of adaptations.

In conclusion, we generated I79N^+/−^ *TNNT2* hiPSC-CMs using CRISPR/Cas9 and optically mapped their physiological features including voltage and Ca^2+^ transients. Our findings indicate that the alteration of AP and Ca^2+^ handling in I79N^+/−^ *TNNT2* hiPSC-CMs is proarrhythmic at higher stimulation frequencies. Remarkably, the transcriptome was dramatically impacted by the insertion of a point mutation in one allele of troponin T and likely has profound consequences in a disease phenotype. The hiPSC-CMs along with the genome-editing tools demonstrate the utility of this technology for modeling sarcomeric mutations linked with HCM, identifying the arrhythmogenic factors and, potentially, developing therapeutic agents for the treatment of HCM.

## Data Availability

The original contributions presented in the study are included in the article/Supplementary Material; further inquiries can be directed to the corresponding author.
