# Supplementary material for: Mechanisms of Arrhythmogenicity of Hypertrophic Cardiomyopathy-Associated Troponin T (TNNT2) Variant I79N
Source: Front Cell Dev Biol. 2021 Dec 17;9:787581. doi: 10.3389/fcell.2021.787581 (PMC8718794; doi:10.3389/fcell.2021.787581)
Supplement: Supplementary file 1 [file DataSheet1.pdf]

Supplemental Figures and Tables Captions

Table S1. Recombinant protein composition of human cardiac reconstituted thin filaments. All cTn and RTF utilized in this study were labeled with IAANS at T53C position with the two additional mutations C35S and C84S.

Table S2. Oligonucleotide sequences for sgRNA, ssODN and PCR primers used for hiPSC genome editing.

Table S3. List of 251 transcripts in the customized NanoString codeset.

Figure S1. Sequence alignment of the partial cTnT protein among different species. A multiple sequence alignment of the partial canonical cTnT protein from human (Uniprot ID: P45379), bovine (Uniprot ID: P13789), rabbit (Uniprot ID: P09741), rat (Uniprot ID: P50753), mouse (Uniprot ID: P50752), chicken (Uniprot ID: P02642), and zebrafish (Uniprot ID: Q90Y46) was performed using Clustal Omega. I79 residues are boxed in red and are highly conserved across different species. The human cTnT sequence shown here includes exon 5 which encodes 10 amino acids, which are boxed in blue. Exon 5 is not included in the splice variant normally expressed in the adult heart and so the residue numbers are shifted by ten in this figure.

Figure S2. Genome-editing to create *TNNT2* I79N$^{+/-}$ hiPSC line. Panel A. Genome editing strategy map in which the relevant portion of the TNNT2 nucleotide sequence is shown. Residue 79 is within exon 9 and the annealing position of the designed single guide RNA (sg RNA) is shown in orange and the PAM site (TGG) in pink. The donor DNA or single-stranded oligodeoxynucleotide (ssODN) spanned the region shown by the brown (36 bp PAM distal) and green (91 bp PAM proximal) arrows. Panel B. Sanger sequencing of relevant nucleotides. The gene edited nucleotide is shown by the red arrow in which the codon for Isoleucine (I) ATC was genome edited to AAC (Asparagine (N)) in one allele rendering a I79N heterozygous mutant. The blue arrow indicates the silent mutation (C->G) that was included in the sgRNA target sequence to prevent CRISPR from re-cutting the target sequence once the desired edit was introduced. The top 10 sites that scored high for off target or low for on-target were selected using "Custom Alt-R® CRISPR-Cas9 guide RNA tool" from IDT. The top 10 off target sites were then Sanger sequenced to ensure that the probability of off target mutations was minimized.

Figure S3. The expression of *TNNI3/TNNI1* in WT and I79N$^{+/-}$ hiPSC-CMs.

Figure S4. Heat map and Principal Component Analysis (PCA) of data from NanoString multiplex gene expression analysis. **Panel A**, Heat map of unsupervised hierarchical clustering of all 251 genes. I79N$^{+/-}$ mutation is clustering away from WT with low variability within group. Patterns ofgene expression are evident through the effect of the mutation. **Panel B** PCA of normalized gene expression for I79N$^{+/-}$ (blue) vs WT (black) hiPSC-CMs shows PC1 and PC2 account for 73% and 16% of the variance, respectively. In PCA, I79N$^{+/-}$ samples are clustered away from WT.

Figure S5. NanoString multiplex gene expression functional analysis. Panels A and B. Top 5 functional pathways (i.e., enrichment term) based on adjusted p-value (< 0.05) across six gene set libraries. Enrichment analysis was conducted using the 45 upregulated and 23 downregulated differentially expressed genes (DEG) in I79N$^{+/-}$ hiPSC-CMs compared to WT hiPSC-CMs. **Panels C and D**. Top 5 enriched Gene Ontology (GO) terms sorted by adjusted p-value. X-axis represents the combined score (combination of the p-value and the z-score). Y-axis refers to the namesof pathways terms split by the gene set libraries. Data point size represents the Odds ratio which is the estimate from Fisher's exact test calculated

through Enrichr (Chen et al., 2013; Kuleshov et al., 2016). Color gradient refers to the adjusted p-value of each term. GO_BP: Gene Ontology biological process, GO_CC: Gene Ontology cellular component, GO_MF: Gene Ontology molecular function, Hu_Pheno: human phenotype, Jensen_Diseases: disease database from Jensen Lab, MGI_Pheno: Mammalian Phenotype Ontology. $I79N^{+/-}$ (n=3) to WT (n=4) TNNT2 hiPSC-CMs.

Table S1.

<table>
<tr><th>Construct</th><th>Gene Name</th><th>Description</th><th>Length</th><th>Molecular Weight (kDa)*</th><th>Extinction Coefficient ($M^{-1}cm^{-1}$)*</th><th>Uniprot ID</th></tr>
<tr><td># WT cTnC^{T53C}</td><td>TNNC1</td><td>Full length TnC with three mutations (T53C, C35S, C84S) for fluorescence labeling specifically at T53C for functional studies.</td><td>161</td><td>18</td><td>4470</td><td>P63316</td></tr>
<tr><td colspan="7">Troponin I and Troponin T Subunits</td></tr>
<tr><td>WT cTnI</td><td>TNNI3</td><td>Full length WT cTnI.</td><td>211</td><td>24</td><td>9970</td><td>P19429</td></tr>
<tr><td>WT cTnT</td><td>TNNT2</td><td>Full length WT cTnT (isoform 3).</td><td rowspan="3">288</td><td rowspan="3">35</td><td rowspan="3">16960</td><td rowspan="3">P45379</td></tr>
<tr><td>I79N cTnT</td><td>TNNT2</td><td>Full length cTnT with I79N mutation.</td></tr>
<tr><td></td><td></td><td></td></tr>
<tr><td colspan="7">Other constructs</td></tr>
<tr><td>Alpha MAS-Tm</td><td>TPM1</td><td>Full-length Tm with the additional tripeptide (Met-Ala-Ser) to the N-terminus to mimic acetylation process in native Tm and ensure its binding to actin filaments.</td><td>287</td><td>33</td><td>8940</td><td>P09493</td></tr>
</table>

Both the molecular weight and extinction coefficients were calculated using ProtParam[1].
All cTn and RTF utilized in this study were labeled with IAANS at T53C position with the two additional mutations C35S and C84S.

[1]Gasteiger, E. et al. Protein Identification and Analysis Tools on the ExPASy Server. *The Proteomics Protocols Handbook*, 571 (2005).

Table S2. Oligonucleotide sequences for sgRNA, ssODN and PCR primers used for hiPSC genome editing

**sgRNA**	
**I79N - F**	5'-CACCGAGTCCACTCTCTCTCCATCG-3'
**I79N - R**	5'- AAACCGATGGAGAGAGAGTGGACTC-3'
**ssODN**	
**I79N**	5' AGTCCCTGGGTCCAGAATGGGGCTGATGCTGACTATTCCTCTCTCCAACAGGTCGTTCATGCCCAACTTGGTGCCTCCCAAGAATCC CGATGGAGAGAGAGTCGACTTTGATGTAAGCGGTGGCTGT -3'
**PCR primers for sequencing**	
**I79 - F**	5'-GCTTCTTTGATTCCAAGTTGTGTG-3'
**I79 - R**	5'-CCCATCCCACCTATGCTC-3'

NanoString Custom Codeset

Table S3.

		Gene Name	Accession no.	Probe Position				Gene name	Accession no.	Probe Position
**1**		ABCC8	NM_000352.4	1761-1860		**33**		CALM3	NM_005184.2	1306-1405
**2**		ACADM	NM_000016.5	1290-1389		**34**		CALR	NM_004343.2	966-1065
**3**		ACTA1	NM_001100.3	46-145		**35**		CaMK2A	NM_171825.1	4081-4180
**4**		B actin	NM_001101.2	1011-1110		**36**		CaMK2B	NM_001220.3	366-465
**5**		ACTC1	NM_005159.4	2006-2105		**37**		CaMK2D	NM_001221.3	1906-2005
**6**		ACTN2	NM_001103.2	413-512		**38**		CaMK2G	NM_001222.2	234-333
**7**		ADRB1	NM_000684.1	796-895		**39**		CASQ2	NM_001232.3	598-697
**8**		ADRB2	NM_000024.3	1246-1345		**40**		CAV3	NM_001234.3	212-311
**9**		AKT	NM_0010144	1276-1375		**41**		CER1	NM_005454.2	589-688
**10**		ALDH1A1	NM_000689.3	12-111		**42**		CHRM2	NM_0010066	1171-1270
**11**		ALDH1A2	NM_003888.2	3131-3230		**43**		CKB	NM_001823.4	321-420
**12**		ALDH1A3	NM_000693.2	2281-2380		**44**		CKM	NM_001824.4	136-235
**13**		CD13	NM_001150.1	2671-2770		**45**		COL1A1	NM_000088.3	5211-5310
**14**		JCTN or	NM_032466.3	396-495		**46**		COL2A1	NM_001844.4	4746-4845
**15**		ATP1A2	NM_000702.3	4041-4140		**47**		COL3A1	NM_000090.3	181-280
**16**		ATP1A3	NM_152296.4	2626-2725		**48**		COL4A2	NM_001846.2	1151-1250
**17**		ATP2A2	NM_001681.3	1397-1496		**49**		COL4A3	NM_000091.4	1085-1184
**18**		ATP5F1	NM_001688.4	646-745		**50**		COL9A2	NM_001852.3	2414-2513
**19**		B2M	NM_004048.2	26-125		**51**		CPT1B	NM_004377.3	1655-1754
**20**		BMP10	NM_014482.1	441-540		**52**		CRHR2	NM_001883.2	236-335
**21**		BMP2	NM_001200.2	1516-1615		**53**		CS	NM_004077.2	741-840
**22**		BMP4	NM_001202.3	660-759		**54**		CTNNB1	NM_0010982	1816-1915
**23**		BRCA1	NM_007305.2	1276-1375		**55**		CXCR4	NM_003467.2	1336-1435
**24**		BTAF1	NM_003972.2	1697-1796		**56**		CYP26A1	NM_057157.2	1591-1690
**25**		BTK	NM_000061.1	571-670		**57**		CYP26B1	NM_019885.2	1806-1905
**26**		CACNA1C	NM_199460.2	4786-4885		**58**		DKK1	NM_012242.2	76-175
**27**		CACNA1D	NM_000720.2	5196-5295		**59**		DYNC1I1	NM_0011355	1183-1282
**28**		CACNA1G	NM_198397.1	1381-1480		**60**		DYNLL2	NM_080677.2	639-738
**29**		CACNA1H	NM_021098.2	3330-3429		**61**		EMILIN2	NM_032048.2	1446-1545
**30**		CACNA2D1	NM_000722.2	2909-3008		**62**		ERBB2	NM_0010058	1256-1355
**31**		CALM1	NM_006888.3	1684-1783		**63**		FGF17	NM_003867.2	280-379
**32**		CALM2	NM_001743.3	869-968		**64**		FGF19	NM_005117.2	787-886

NanoString Custom Codeset

		Gene name	Accession number	Probe Position			Gene Name	Accession Number	Probe Position
**65**		FGF2	NM_002006.4	621-720	**97**		HOPX	NM_139212.3	511-610
**66**		FGF4	NM_002007.2	689-788	**98**		HRC	NM_002152.2	367-466
**67**		FGF8	NM_033163.3	545-644	**99**		IPO8	NM_006390.2	861-960
**68**		PKBP1B or FKBP12.6	NM_000801.4	41-140	**100**		IRX4	NM_016358.2	359-458
**69**		FLNA	NM_001456.3	7336-7435	**101**		ISL1	NM_002202.2	1376-1475
**70**		FLNB	NM_001164317.1	2125-2224	**102**		ISL2	NM_145805.1	109-208
**71**		FOXA1	NM_004496.2	2466-2565	**103**		JAG1	NM_000214.2	916-1015
**72**		FOXF1	NM_001451.2	1761-1860	**104**		JAK1	NM_002227.1	286-385
**73**		FOXH1	NM_003923.2	696-795	**105**		JAK2	NM_004972.3	1465-1564
**74**		GAPDH	NM_001256799.1	387-486	**106**		JAK3	NM_000215.2	1716-1815
**75**		GATA2	NM_032638.3	1496-1595	**107**		JPH2	NM_020433.4	525-624
**76**		GATA4	NM_002052.3	2141-2240	**108**		KCNA4	NM_002233.3	657-756
**77**		GATA6	NM_005257.3	2131-2230	**109**		KCNA5	NM_002234.2	2331-2430
**78**		GJA1	NM_000165.3	706-805	**110**		KCND3	NM_004980.4	1011-1110
**79**		GJA5	NM_005266.5	1993-2092	**111**		KCNH2	NM_172057.2	781-880
**80**		GJC1	NM_001080383.1	811-910	**112**		KCNJ2	NM_000891.2	621-720
**81**		GNAI1	NM_002069.4	671-770	**113**		KCNJ3	NM_001260508.1	429-528
**82**		GNAI2	NM_002070.2	1001-1100	**114**		KCNJ5	NM_000890.3	631-730
**83**		GNAI3	NM_006496.1	1151-1250	**115**		KCNJ8	NM_004982.2	881-980
**84**		GNAO1	NM_020988.2	1423-1522	**116**		KCNK1	NM_002245.3	721-820
**85**		GNAS	NM_080425.1	1911-2010	**117**		KCNK3	XM_005264293.1	667-766
**86**		GREM2	NM_022469.3	3006-3105	**118**		KCNK5	NM_003740.3	3616-3715
**87**		GYPA	NM_002099.4	2312-2411	**119**		KCNN1	NM_002248.3	703-802
**88**		HAND1	NM_004821.2	1231-1330	**120**		KCNN2	NM_021614.2	1893-1992
**89**		HAND2	NM_021973.2	1822-1921	**121**		KCNN3	NM_002249.4	2636-2735
**90**		HAS2	NM_005328.2	961-1060	**122**		KCNQ1	NM_181798.1	836-935
**91**		HCN1	NM_021072.2	1451-1550	**123**		KDR	NM_002253.2	1421-1520
**92**		HCN2	NM_001194.3	2075-2174	**124**		KlF20A	NM_005733.2	1210-1309
**93**		HCN4	NM_005477.2	4911-5010	**125**		KIT	NM_000222.1	6-105
**94**		HEY1	NM_012258.3	586-685	**126**		KLF4**	NM_004235.4	1981-2080
**95**		HEY2	NM_012259.2	1576-1675	**127**		KMT2A	NM_005933.2	14001-14100
**96**		HIF1A	NM_001530.2	1986-2085	**128**		KMT2B	NM_014727.1	7846-7945

NanoString Custom Codeset

		Gene Name	Accession Number	Probe Position				Gene	Accession	Probe Position
**129**		KMT2C	NM_170606.2	11974-12073		**161**		NR2F1	NM_005654.4	3111-3210
**130**		KMT2D	NM_003482.3	6071-6170		**162**		NR2F2	NM_021005.2	1531-1630
**131**		LDHA	NM_001165414.1	1691-1790		**163**		ORAI1	NM_032790.3	911-1010
**132**		LEFTY1	NM_020997.2	1406-1505		**164**		PDGFRA	NM_006206.3	1926-2025
**133**		LIN28	NM_024674.4	1961-2060		**165**		PDK2	NM_002611.3	436-535
**134**		JNK3	NM_138981.2	451-550		**166**		PDK4	NM_002612.3	1676-1775
**135**		JNK1-alpha	NM_002750.2	946-1045		**167**		p110a	NM_006218.2	2446-2545
**136**		JNK2-alpha	XM_005265940.1	164-263		**168**		PITX2	NM_000325.5	1382-1481
**137**		MEF2c	NM_002397.3	2446-2545		**169**		PKP2	NM_001005242.2	1692-1791
**138**		MESP1	NM_018670.3	865-964		**170**		PLCB1	NM_182734.1	171-270
**139**		MIXL1	NM_031944.1	419-518		**171**		PLCB2	NM_004573.2	411-510
**140**		IP3R	NM_130385.2	3056-3155		**172**		PLCD1	NM_006225.3	2126-2225
**141**		MSX2	NM_002449.4	31-130		**173**		PLCD3	NM_133373.4	1308-1407
**142**		MYBPC3	NM_000256.3	3429-3528		**174**		PLCE1	NM_001165979.1	393-492
**143**		MYC**	NM_002467.3	1611-1710		**175**		PLCG1	NM_002660.2	2291-2390
**144**		MYH6	NM_002471.3	928-1027		**176**		PLCG2	NM_002661.2	526-625
**145**		MYH7	NM_000257.2	1917-2016		**177**		PLN	NM_002667.3	213-312
**146**		MYL2	NM_000432.3	721-820		**178**		POLR2A	NM_000937.2	3776-3875
**147**		MYL7	XM_005249817.2	339-438		**179**		POU3F4	NM_000307.3	78-177
**148**		MYO1E	NM_004998.2	4556-4655		**180**		POU5F1	NM_002701.4	1226-1325
**149**		NANOG**	NM_024865.2	1101-1200		**181**		PPIA	NM_021130.3	316-415
**150**		NCAM1	NM_000615.5	1621-1720		**182**		PPP1CA	NM_002708.3	506-605
**151**		NFATC4	NM_001136022.2	2297-2396		**183**		PPP2CA	NM_002715.2	1076-1175
**152**		NKX2-5	NM_004387.3	219-318		**184**		PPP3CB	NM_021132.2	271-370
**153**		NNT	NM_012343.3	2431-2530		**185**		PRKACA	NM_002730.3	401-500
**154**		NODAL	NM_018055.3	321-420		**186**		PRKACB	NM_182948.2	806-905
**155**		NOTCH1	NM_017617.3	8212-8311		**187**		PRKACG	NM_002732.2	776-875
**156**		NOTCH2	NM_024408.3	2843-2942		**188**		PRKAR1A	NM_212472.1	368-467
**157**		NOTCH3	NM_000435.2	1966-2065		**189**		PRKAR1B	NM_001164759.1	1113-1212
**158**		NOTCH4	NM_004557.3	6421-6520		**190**		PRKAR2A	NM_004157.2	479-578
**159**		NPPA	NM_006172.2	116-215		**191**		PRKAR2B	NM_002736.2	1351-1450
**160**		NPPB	NM_002521.2	497-596		**192**		PRKCA	NM_002737.2	681-780

NanoString Custom Codeset

		Gene Name	Accession no.	Probe Position				Gene Name	Accession no.	Probe Position
**193**		PRKCB	NM_212535.1	1751-1850		**225**		T	NM_003181.2	1837-1936
**194**		PRKCG	NM_002739.3	446-545		**226**		TEC	NM_003215.1	1231-1330
**195**		PKG1	NM_006258.3	995-1094		**227**		CD90	NM_006288.2	136-235
**196**		PTGS2	NM_000963.1	496-595		**228**		TNNI1	NM_003281.3	976-1075
**197**		RARA	NM_001033603.1	1191-1290		**229**		TNNI3	NM_000363.4	226-325
**198**		RARB	NM_000965.3	2756-2855		**230**		TNNT2 exon 5	NM_000364.3	132-231
**199**		RARG	NM_000966.3	1541-1640		**231**		TNNT2	NM_001276346.1	797-896
**200**		RBM20	NM_001134363.1	6943-7042		**232**		TOP2A	NM_001067.3	3564-3663
**201**		ROR2	NM_004560.2	736-835		**233**		TPM1	NM_000366.5	843-942
**202**		RPS13	NM_001017.2	332-431		**234**		TRDN	NM_001251987.1	297-396
**203**		RYR2	NM_001035.2	1184-1283		**235**		TRH	NM_007117.2	1883-1982
**204**		SCN4A	NM_000334.4	6787-6886		**236**		TRPC1	NM_003304.4	991-1090
**205**		SCN5A	NM_198056.2	8341-8440		**237**		TRPC2	NR_002720.2	561-660
**206**		SGK1	NM_005627.2	1791-1890		**238**		TRPC3	NM_001130698.1	1479-1578
**207**		SHOX2	NM_003030.3	830-929		**239**		TRPC4	NM_001135956.1	2436-2535
**208**		SIRPA	NM_080792.2	3116-3215		**240**		TRPC5	NM_012471.2	3686-3785
**209**		SLC16A1	NM_003051.3	636-735		**241**		TRPC6	NM_004621.5	1001-1100
**210**		SLC2A1/GLUT1	NM_006516.2	2501-2600		**242**		TRPC7	NM_001167576.1	1119-1218
**211**		SLC2A4/GLUT4	NM_001042.2	2121-2220		**243**		TSHR	NM_001018036.2	736-835
**212**		SLC8A1	NM_021097.1	441-540		**244**		SSEA5	NM_001270483.1	1007-1106
**213**		SLN	NM_003063.2	376-475		**245**		TTN b	NM_003319.4	1805-1904
**214**		SOX2	NM_003106.2	152-251		**246**		TTN	XM_024453094.1	33791-33890
**215**		SPCS1	NM_014041.3	611-710		**247**		VCAM-1	NM_001078.3	2536-2635
**216**		STIM1	NM_003156.3	3101-3200		**248**		VEGFA	NM_001025366.1	1326-1425
**217**		TACR1	NM_015727.1	856-955		**249**		WNT3	NM_030753.3	1336-1435
**218**		TACR3	NM_001059.1	471-570		**250**		WNT3A	NM_033131.2	699-798
**219**		TBX1	NM_080646.1	541-640		**251**		ZFP42 (or REX1)	NM_174900.3	946-1045
**220**		TBX18	NM_001080508.2	545-644						
**221**		TBX2	NM_005994.3	936-1035						
**222**		TBX20	NM_020417.1	661-760						
**223**		TBX3	NM_005996.3	3276-3375						
**224**		TBX5	NM_080718.1	246-345						

Figure S1.

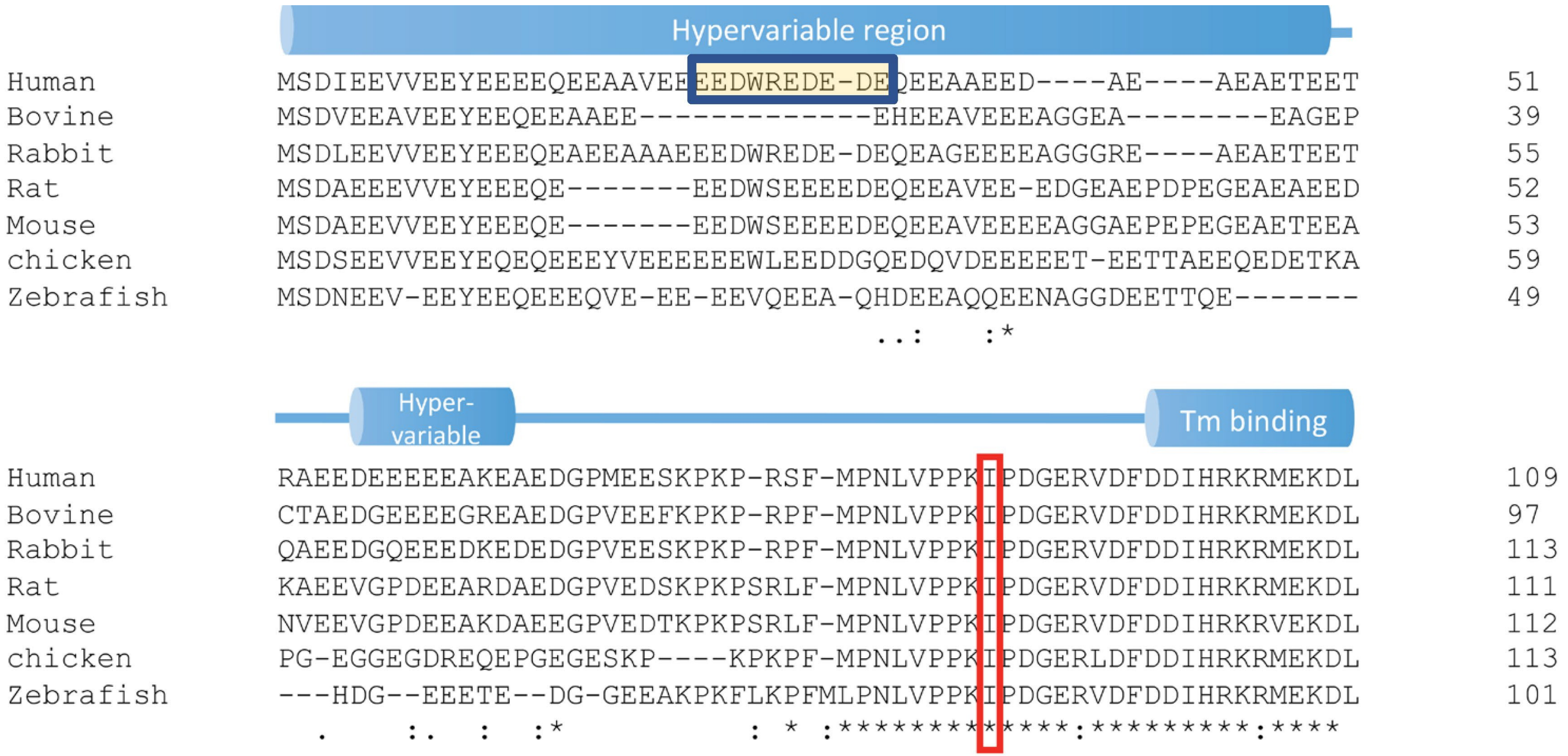
Hypervariable region
Human MSDIEEVVEEYEEEEQEEAAVEEEEDWREDE-DEDEEAAEED----AE----AEAETEET 51
Bovine MSDVEEAVEEYEEQEEAAEE------------EHEEAVEEEAGGEA--------EAGEP 39
Rabbit MSDLEEVVEEYEEEQEAEEAAAEEEDWREDE-DEQEAGEEEEAGGGRE----AEAETEET 55
Rat MSDAEEEVVEYEEEQE-------EEDWSEEEEDEQEEAVEE-EDGEAEPDPEGEAEAEED 52
Mouse MSDAEEVVEEYEEEQE-------EEDWSEEEEDEQEEAVEEEEAGGAEPEPEGEAETEEA 53
chicken MSDSEEVVEEYEQEQEEEYVEEEEEEWLEEDDGQEDQVDEEEEET-EETTAEEQEDETKA 59
Zebrafish MSDNEEV-EEYEEQEEEQVE-EE-EEVQEEA-QHDEEAQQEENAGGDEETTQE------- 49
..: :*
Hyper-variable
Tm binding
Human RAEEDEEEEEAKEAEDGPMEESKPKP-RSF-MPNLVPPKIPDGERVDFDDIHRKRMEKDL 109
Bovine CTAEDGEEEEGREAEDGPVEEFKPKP-RPF-MPNLVPPKIPDGERVDFDDIHRKRMEKDL 97
Rabbit QAEEDGQEEEDKEDEDGPVEESKPKP-RPF-MPNLVPPKIPDGERVDFDDIHRKRMEKDL 113
Rat KAEEVGPDEEARDAEDGPVEDSKPKPSRLF-MPNLVPPKIPDGERVDFDDIHRKRMEKDL 111
Mouse NVEEVGPDEEAKDAEEGPVEDTKPKPSRLF-MPNLVPPKIPDGERVDFDDIHRKRVEKDL 112
chicken PG-EGGEGDREQEPGEGESKP----KPKPF-MPNLVPPKIPDGERLDFDDIHRKRMEKDL 113
Zebrafish ---HDG--EEETE--DG-GEEAKPKFLKPFMLPNLVPPKIPDGERVDFDDIHRKRMEKDL 101
. :. : :* : * :*************:*********:****

Figure S2.

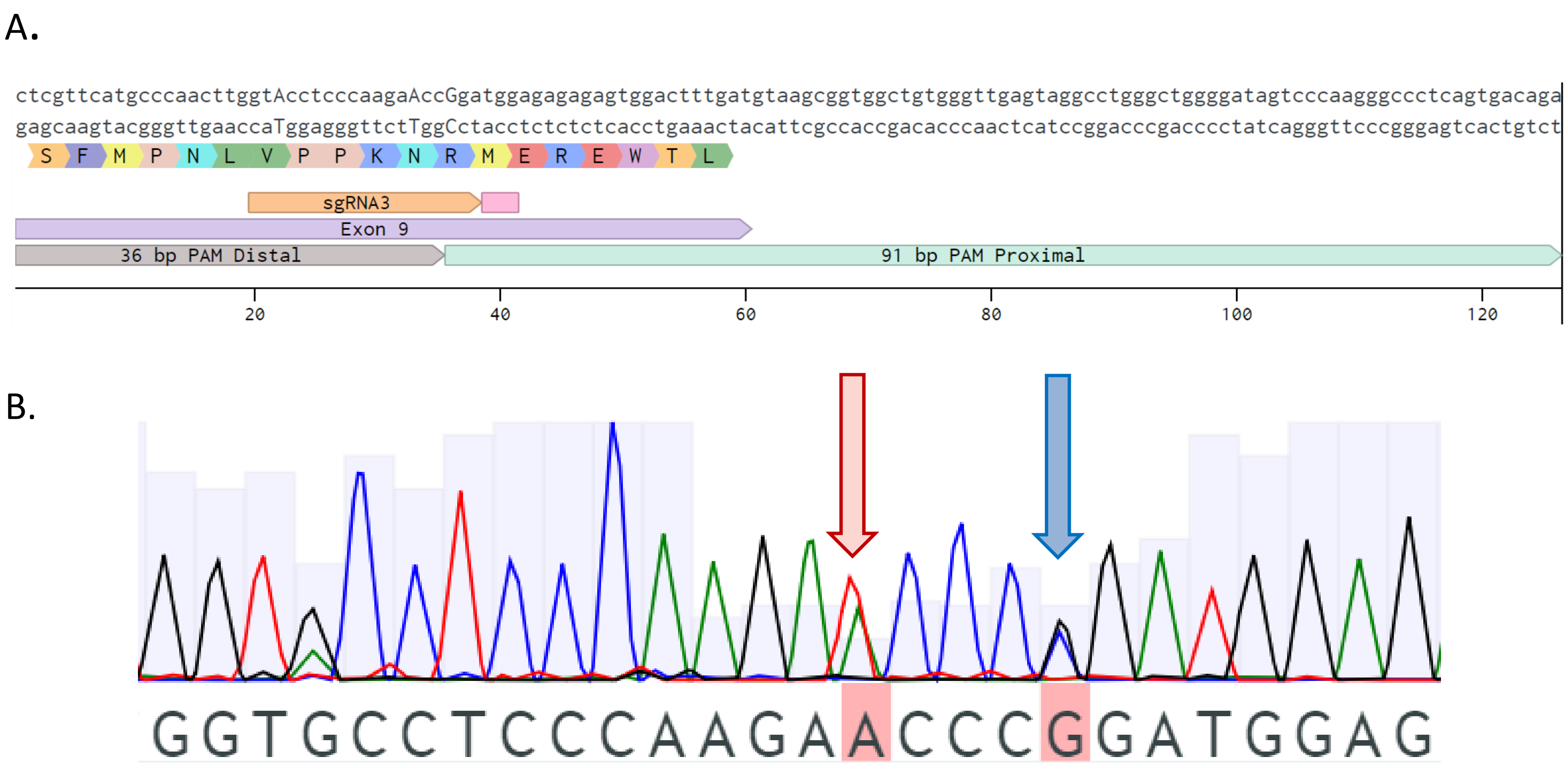
A.
ctcgttcatgcccaacttggtAcctcccaagaAccGgatggagagagagtggactttgatgtaagcggtggctgtgggttgagtaggcctgggctggggatagtcccaagggccctcagtgacaga
gagcaagtacgggttgaaccaTggagggttctTggCctacctctctctcacctgaaactacattcgccaccgacacccaactcatccggacccgacccctatcagggttcccgggagtcactgtct
S F M P N L V P P K N R M E R E W T L
sgRNA3
Exon 9
36 bp PAM Distal
91 bp PAM Proximal
20
40
60
80
100
120
B.
GGTGCCTCCCAAGAACCCCGGATGGAG

Figure S3.

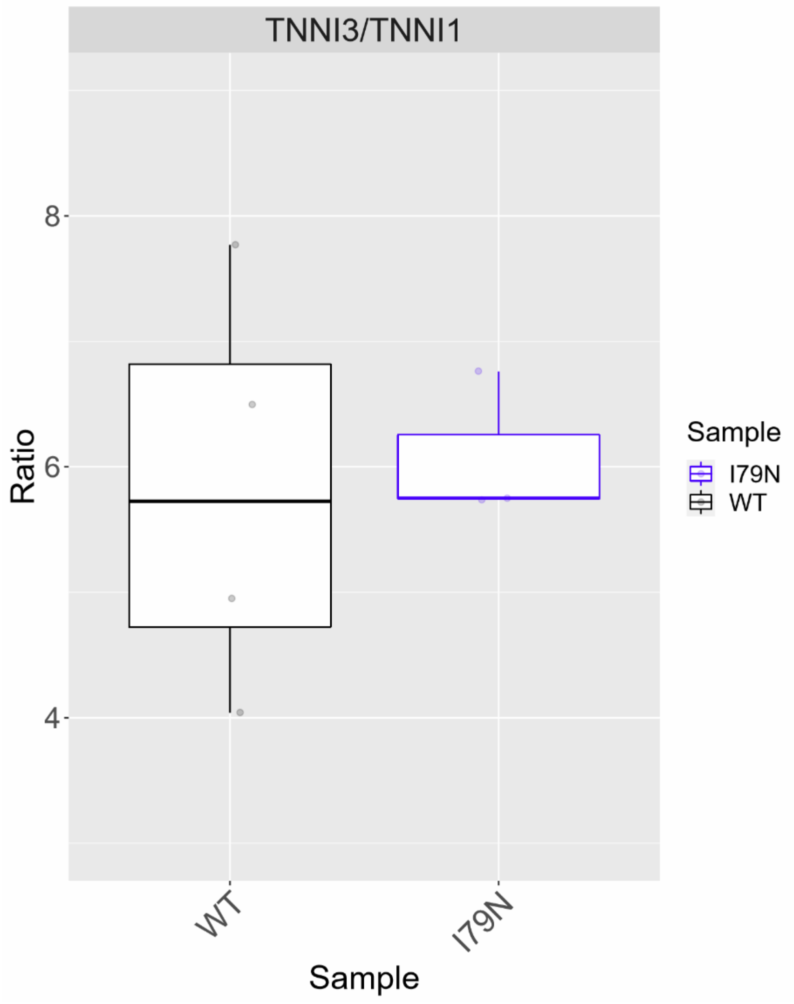

Figure S4.

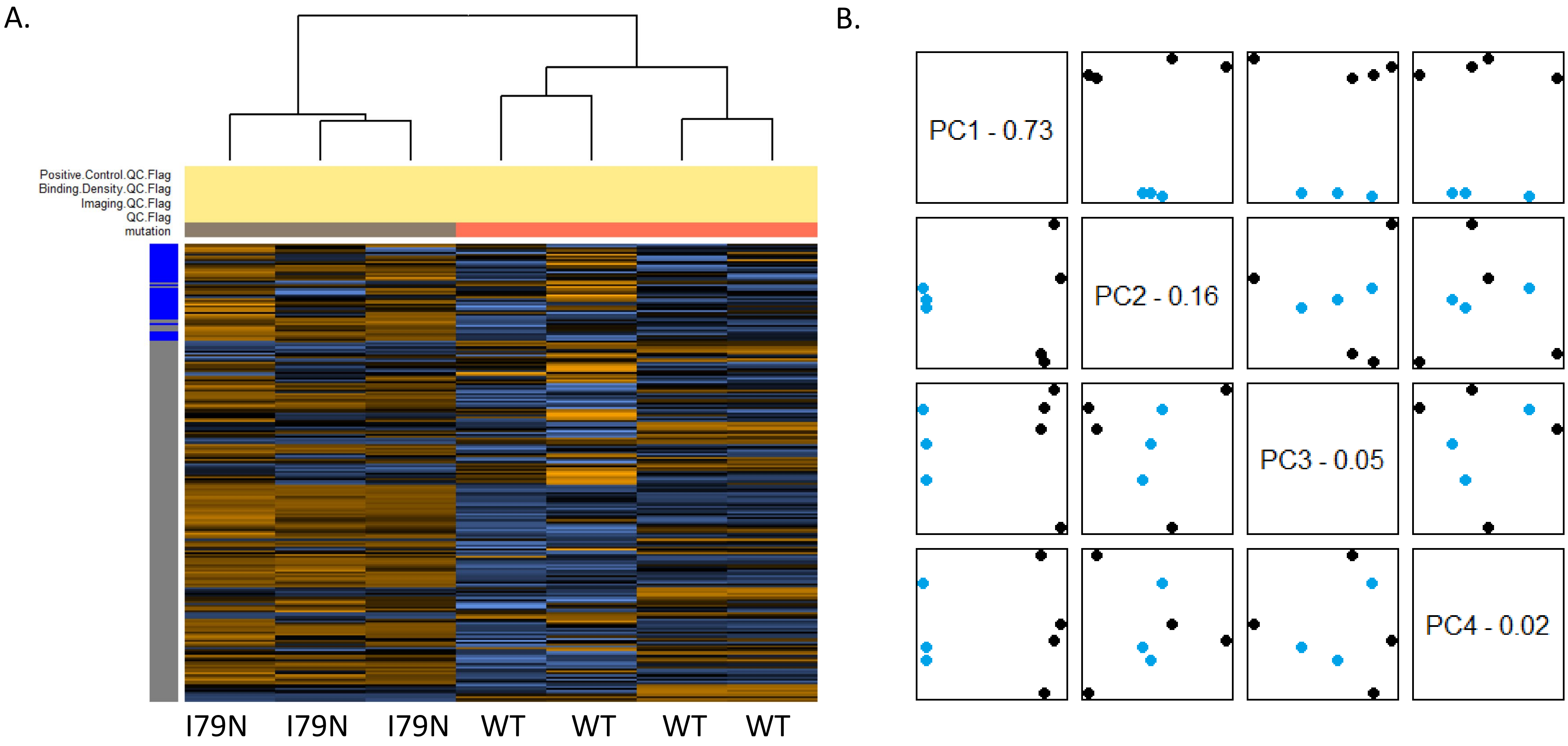
A.
Positive.Control.QC.Flag
Binding.Density.QC.Flag
Imaging.QC.Flag
QC.Flag
mutation
I79N
I79N
I79N
WT
WT
WT
WT
B.
PC1 - 0.73
PC2 - 0.16
PC3 - 0.05
PC4 - 0.02

Figure S5

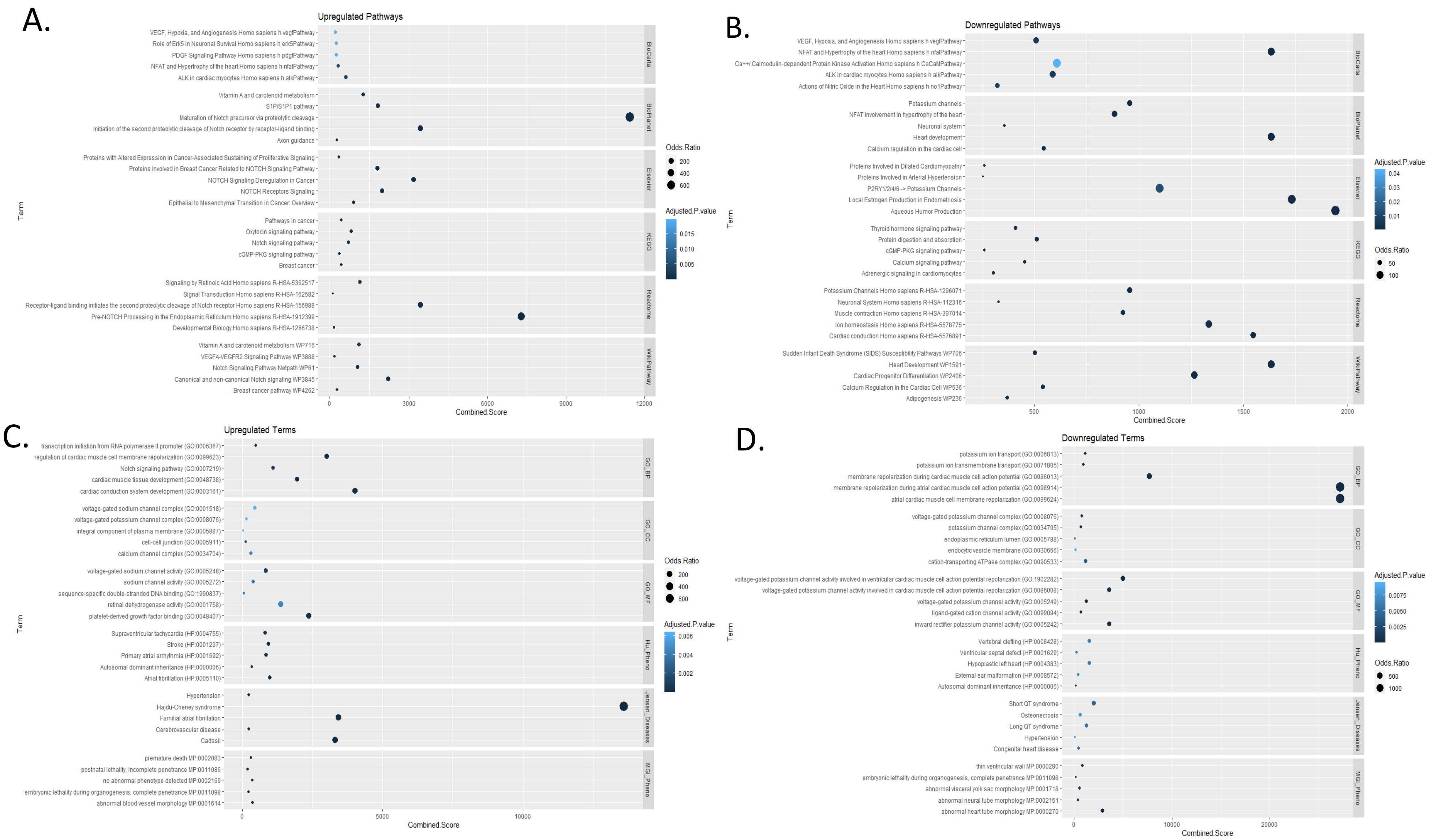
A.
Upregulated Pathways
B.
Downregulated Pathways
C.
Upregulated Terms
D.
Downregulated Terms
Combined.Score
Term
Odds.Ratio
Adjusted.P.value
